# Generation and validation of a formula to calculate hemoglobin loss on a cohort of healthy adults subjected to controlled blood loss

**DOI:** 10.1186/s12967-021-02783-9

**Published:** 2021-03-20

**Authors:** Max Hahn-Klimroth, Philipp Loick, Soo-Zin Kim-Wanner, Erhard Seifried, Halvard Bonig

**Affiliations:** 1 Goethe University Mathematics Institute, Frankfurt, Germany; 2 German Red Cross Blood Service BaWüHe, Institute Frankfurt, Sandhofstraße 1, 60528 Frankfurt, Germany; 3 Institute for Transfusion Medicine and Immunohematology, Goethe University, Frankfurt, Germany; 4 Department of Medicine/Hematology, University of Washington, Seattle, WA USA

**Keywords:** Surgical blood loss, Blood management, Blood loss calculator, Blood loss formula, Anemia management, Machine learning

## Abstract

**Background:**

The ability to approximate intra-operative hemoglobin loss with reasonable precision and linearity is prerequisite for determination of a relevant surgical outcome parameter: This information enables comparison of surgical procedures between different techniques, surgeons or hospitals, and supports anticipation of transfusion needs. Different formulas have been proposed, but none of them were validated for accuracy, precision and linearity against a cohort with precisely measured hemoglobin loss and, possibly for that reason, neither has established itself as gold standard. We sought to identify the minimal dataset needed to generate reasonably precise and accurate hemoglobin loss prediction tools and to derive and validate an estimation formula.

**Methods:**

Routinely available clinical and laboratory data from a cohort of 401 healthy individuals with controlled hemoglobin loss between 29 and 233 g were extracted from medical charts. Supervised learning algorithms were applied to identify a minimal data set and to generate and validate a formula for calculation of hemoglobin loss.

**Results:**

Of the classical supervised learning algorithms applied, the linear and Ridge regression models performed at least as well as the more complex models. Most straightforward to analyze and check for robustness, we proceeded with linear regression. Weight, height, sex and hemoglobin concentration before and on the morning after the intervention were sufficient to generate a formula for estimation of hemoglobin loss. The resulting model yields an outstanding R^2^ of 53.2% with similar precision throughout the entire range of volumes or donor sizes, thereby meaningfully outperforming previously proposed medical models.

**Conclusions:**

The resulting formula will allow objective benchmarking of surgical blood loss, enabling informed decision making as to the need for pre-operative type-and-cross only vs. reservation of packed red cell units, depending on a patient’s anemia tolerance, and thus contributing to resource management.

**Supplementary Information:**

The online version contains supplementary material available at 10.1186/s12967-021-02783-9.

## Background

Blood, or, more precisely, hemoglobin (Hb) loss during surgery is a risk factor for the requirement of a red blood cell (RBC) transfusion; blood loss is also a quality indicator for medical or surgical procedures [1–12]. Surgical (i.e. functional) success of a procedure provided, hemoglobin loss is thus one of the most salient outcome parameters of the quality of an intervention, whether for comparison of competing techniques, of hospitals, or of individual surgeons. Once the typical blood loss during a certain intervention has been established with some robustness, use of that value and a specific patient’s predicted individual anemia tolerance can be used to make decisions with respect to perioperative anemia management–will type and cross be required, will it be sufficient, or should RBC products be immediately available in the operating room–and can thus aid blood bank inventory management. While hemoglobin management and hemoglobin loss as a critical quality-defining outcome parameter are firmly established, useful, practical tools for assessment of intra-operative hemoglobin loss are lacking [13, 14]. Methods to gauge hemoglobin loss into surgical gauze, using photometric methods, were already proposed in the 1950s [15]. A number of simple formulas drawing on pre- and post-operative hemoglobin or hematocrit (Hct), typically incorporating total blood volume/RBC volume/ hemoglobin mass of the respective patient, have been proposed since [16–22]. None of these caught on, presumably because the former is cumbersome and the latter poorly validated, and a need to provide techniques for more accurate blood loss quantification was identified [14, 23]. The ascent of “big data” mathematical brute-force technologies as well as the ubiquity of handheld minicomputers which can accommodate complex estimation algorithms now allowed development of a refined formula for hemoglobin loss based on a large cohort of healthy individuals subjected to controlled hemoglobin loss. We identify a minimum dataset required for intraoperative hemoglobin loss calculation across a wide range equivalent to 0.5–4.5 RBC units, demonstrate insufficiency of established formulas with respect to accuracy and linearity, and propose a mathematically relatively complex new formula which is easily applied by entering the minimal data into a software application (web-app) or tabular calculation datasheet.

## Donors and methods

The subjects of the controlled bleed were healthy allogeneic bone marrow donors. All had undergone extensive health assessment according to criteria exceeding donor assessment guidelines of JACIE and WMDA as well as European and national law as previously described [24] and were found healthy except for minor non-limiting conditions such as arterial hypertension, hyperlipidemia, asthma/allergies or hypothyroidism which were either medically controlled or did not require medication. Under general anesthesia, bone marrow was aspirated as described [25]; the total hemoglobin contained in the bone marrow product represents the “controlled hemoglobin loss”. Target volume was dictated by required stem cell dose, although aspiration volume could be limited by donor weight where the theoretical target volume exceeded 1.5% of body weight. During the marrow harvest donors received crystalline replacement fluid of approximately 1 L/h. Collection of one liter of bone marrow takes approximately 45 min. Complete blood count (CBC) and differential were measured on the day before marrow collection. Bone marrow was aspirated transcutaneously from the posterior iliac crest, i.e. unlike during most surgeries the procedure is not associated with unaccounted blood loss and can therefore be measured quite accurately. Bone marrow volume was gauged by weighing the product with precision scales and correcting for density according to hematocrit. Hemoglobin loss (g) was determined as the hemoglobin concentration (g/L) in the bone marrow product times its volume. After awaking from the anesthesia donors were offered liquids by mouth ad libitum and mobilized immediately. Donors were routinely discharged 24–28 h after the marrow harvest; post-procedure CBC was collected immediately prior to discharge. CBC in blood before and after the harvest and in bone marrow were measured with the same Sysmex XT1800 hemacytometer (Norderstedt, Germany) in our accredited laboratory which participates in quality circles; at least three daily controls ensure precision and accuracy of the measurements. The following data were collected and considered for the analyses: Donor height, weight, age, sex and BMI, CBC before and after in blood, CBC and volume in the product.

### Mathematical analyses

The formula was generated by standard machine learning approaches. We strove to employ a broad range of supervised learning algorithms that would stress different aspects of the data, i.e. linear vs. non-linear relationships between independent and predicted variable, parametric vs. non-parametric models. Thus during a model selection phase we considered the following classical supervised learning algorithms.

- Linear regression [26]
- Ridge regression with penalty factor α = 5 [27]
- AdaBoost with decision tree base learner and 100 estimators [28]
- Random Forest with 40 decision trees each with max depth of 4 [29, 30]
- Support vector regression with RBF kernel, C = 9 and ε = 0.9 [31, 32]
- K-nearest neighbors with k = 20 [32, 33]
- Neural network with 3 hidden layers, rectified linear unit (ReLU) activation function and the adam optimizer [34]

The key idea behind model selection in machine learning is to dispassionately try different models on the raw input data independent of the (medical) meaning of individual values, as opposed to classical statistical analysis with hypothesis-driven construction of relationships which can, by necessity, only reinforce known or suspected relationships. Not until after one or more promising models are identified, these are optimized as best as possible on the given data at which point knowledge about the meaning of the identified values can be integrated. Before running the machine learning algorithms we (partially) log-transformed the data, de-correlated some variables (HB_pre and HB_post) by differentiating them and eventually standard scaled all variables (deduct mean and divide by standard deviation) since some algorithms such as kNN require pre-standardization. Once we had settled on the regression model, we performed the analysis without standardization since such preprocessing is not needed for (most) linear models and we wanted to ensure interpretability of the resulting coefficients. With respect to decision trees, while they are mostly used for classification problems, they can be readily adjusted for regression problems. We typically use information gain (maximum reduction in weighted entropy to decide on the splitting attribute) in classification problems; by contrast, the mean squared error is the metric of choice for regression problems using decision trees. All necessary computations were performed in Python 3.8 by using the scikit learn application programming interface (API) [35, 36]. With respect to the data that proved useful, the approach ended up not being very different than what has been used previously, calculation of Hb loss from Hb dilution in blood, i.e. the result of rapid correction of blood volume after a blood loss. Our data representing a wide range of Hb loss values, this uniquely facilitated modeling the function to the data and thus generating the coveted linearity for our formula.

### Ethics statement

This non-interventional retrospective analysis leverages on anonymous outcome data assembled for the legally mandated annual product quality report of our unit. The Ethics Committee of Goethe University Medical School has confirmed that neither specific donor consent nor approval of the Ethics Committee is required.

## Results

### Donors, blood loss

478 healthy donors were subjected to bone marrow aspiration and data were recorded in the donor database. Data from 77 thereof were missing post-collection CBC. Only complete data sets were used; the controlled bleed cohort thus consisted of 401 adult donors, two-thirds male, with a mean age of 28 years (IQR 24–40 years, max. 60 years), mean height of 178 cm (IQR 170–184 cm), mean weight of 79 kg (IQR 67–89 kg) and mean BMI of 24.7 kg/m^2^ (IQR 22.3–27.4 kg/m^2^), i.e. 2%, 33% and 14% were underweight, overweight and obese, respectively. Total hemoglobin in the product ranged from 29–233 g (mean 113 g, IQR 88–141 g), i.e. the equivalent of 0.5–4.5 units of RBCs and thus representing a meaningful range of volumes for many procedures except some relatively bloodless or very large surgeries. No RBC transfusions were administered.

### Determination of minimal data sets and development of optimal prediction algorithms:

The algorithm would be considered “optimal” if the least number of most easily available parameters yielded correct values across the entire range of blood loss volumes or donor sizes and inclusion of further values did not meaningfully improve the prediction, e.g. by narrowing its precision.

The raw dataset features information on the *weight* (kg), the *height* (cm), the *sex*, the *age* (years) and a number of measurements pre- and post-surgery of 401 donors with the corresponding observed *HB loss* (g) harvested into the BM product bag. To be precise, the pre- and post-surgery measurements encompass the hemoglobin concentration (g/L) (denoted *HB_pre* and *HB_post*) and hematocrit concentration (%) (HCT_pre and HCT_post). Moreover, pre-surgery thrombocytes (#/μL) (PLT_pre) and leucocytes (#/μL) (LEUCO_pre) were at our disposal.

**Fig. 1:**
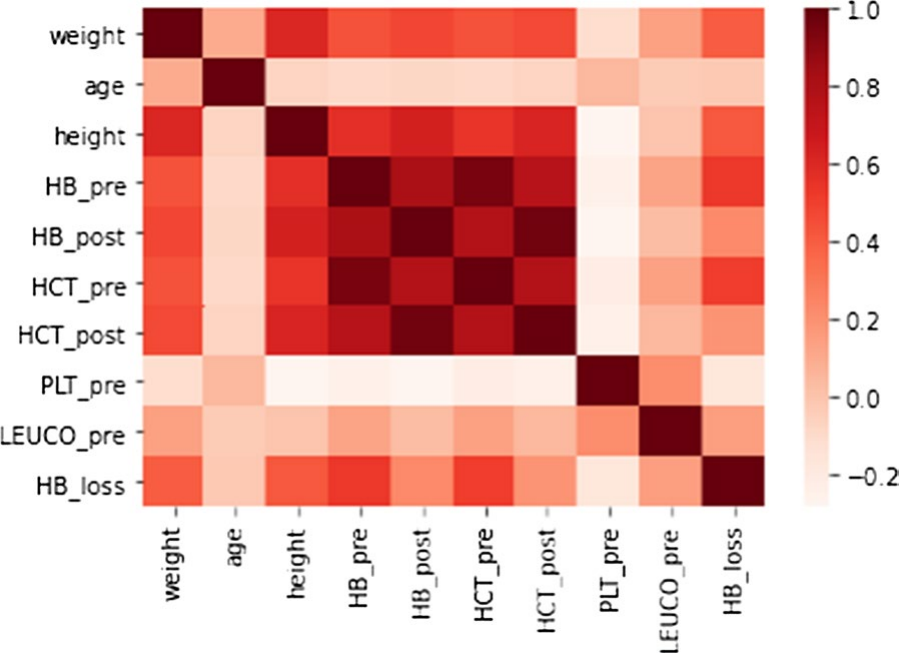
Correlation matrix: Strength of correlations for variables including Hb loss to evaluate the need for transformation of the input data

We set out to develop a machine learning model to predict blood loss from the raw data by training the algorithm against the observed hemoglobin loss. A standard medical approach towards a problem of this nature would be to employ Nadler’s equation [37] to estimate a patient’s blood volume from the patient’s *weight*, *height* and *sex*. In combination with the difference of *HB_pre* and *HB_post* one can obtain an estimate of the hemoglobin loss which is essentially what the Meunier formula suggests [17]. Regressing the actual blood loss against this estimate yields an *R*^2^ value of 47.6%. This approach, close to the Meunier formula [17], will serve as our reference model. Since a standard normal distribution of the input variables and only moderate correlations between variables is desirable for certain supervised learning algorithms, we first investigated the distribution of the input variables and considered the correlation matrix. The former analysis suggested that *weight, age, BMI* and *LEUCO_pre* needed to be log-transformed. The correlation matrix based on these log-transformations in Fig. 1 indicates a particularly strong correlation between the pre- and post-surgery measurements (81% Pearson correlation coefficient between *HB_pre* and *HB_post,* 78% between *HCT_pre* and *HCT_post* and 95% and 97% for the pre- and post-surgery Hb and Hct values respectively) and strong correlations between *height*, *weight* and *sex*. We addressed the former correlations by introducing the auxiliary variable *HB_diff* defined as the difference between *HB_pre* and *HB_post* and in exchange dropping the variable *HB_post*. Hct values were decorrelated from Hb values and a corresponding auxiliary variable *HCT_diff* introduced*.* Regarding the latter correlations, we kept the variables as-is, but would pay close attention to potential multicollinearity issues in the resulting regression model. We followed a standard machine learning approach for model selection, that is, we initially split off a test dataset containing 25% of the samples. Subsequently, we performed a fivefold cross-validation grid search on the remaining train dataset for hyperparameter tuning. For the linear regression model, we dropped all variables that were not significant at a 5% threshold on the train dataset in an effort to design a simple model. Moreover, to emulate the relationship between the Hb concentration difference and the blood volume in determining hemoglobin loss we introduced an interaction term between estimated blood volume as calculated by the Nadler formula and *HB_diff.* Prediction accuracy was measured by the mean squared error (MSE) and the explained variance (EV). With *n* denoting the sample size, *y* = *(y*_*i*_*)*_*i*_* ∈ *_*[n]*_ the vector of true blood loss and *ŷ* = *(ŷ*_*i*_*)*_*I*_* ∈ *_*[n]*_ the predicted blood loss, the formulas for the performance metrics are given by

$$MSE = \frac{{\sum\limits_{i = 1}^n {{{({{\hat y}_i} - {y_i})}^2}} }}{n} \quad EV = 1 - \frac{{Var(y - \hat y)}}{{Var(y)}}$$

Clearly, the smaller the MSE and the larger the EV, the better is the model.

Table 1 summarizes the MSE and EV for the three best-performing supervised learning models given in the previous section and the medical reference model on the test dataset. The fairly simple regression models performed meaningfully better than more advanced techniques such as neural networks. As the linear regression performed on par with Ridge regression, but is more straightforward to analyze and check for robustness, we proceeded with this model. The linear model yields an *R*^2^ of 53.2% thereby meaningfully outperforming the standard medical model. Due to the tractability of the ordinary least-squares-regression model, we were able to put forth simple formulas for women and men to predict hemoglobin loss (coefficients rounded to 3 decimals).

**Table 1:** Prediction accuracy for different statistical models

Model	Test MSE	Test EV
Reference model	696.0	0.542
Linear regression	614.2	0.601
Ridge regression	612.8	0.595
Neural networks	748.6	0.542
Support vector regression	656.9	0.548

Prediction accuracy for the different statistical models is shown. The lower the mean squared error (MSE) and the higher the explained variance (EV), the better the prediction accuracy. Linear and ridge regression outperform the alternative models

$${HB}_{female}=42.212+\left(0.160*{height}^{3}+0.015*weight+0.083\right)*\left({HB}_{pre}-{HB}_{post}\right)$$

$${HB}_{male}=61.767+\left(0.165*{height}^{3}+0.015*weight+0.272\right)*\left({HB}_{pre}-{HB}_{post}\right)$$

(HB_female/male_ [hemoglobin loss in g], height [body height in m], weight [body weight in kg], HBpre/post [hemoglobin concentration before/after controlled hemoglobin loss in g/L]). An excel worksheet implementing these formulae is provided as Additional file 1.

**Fig. 2:**
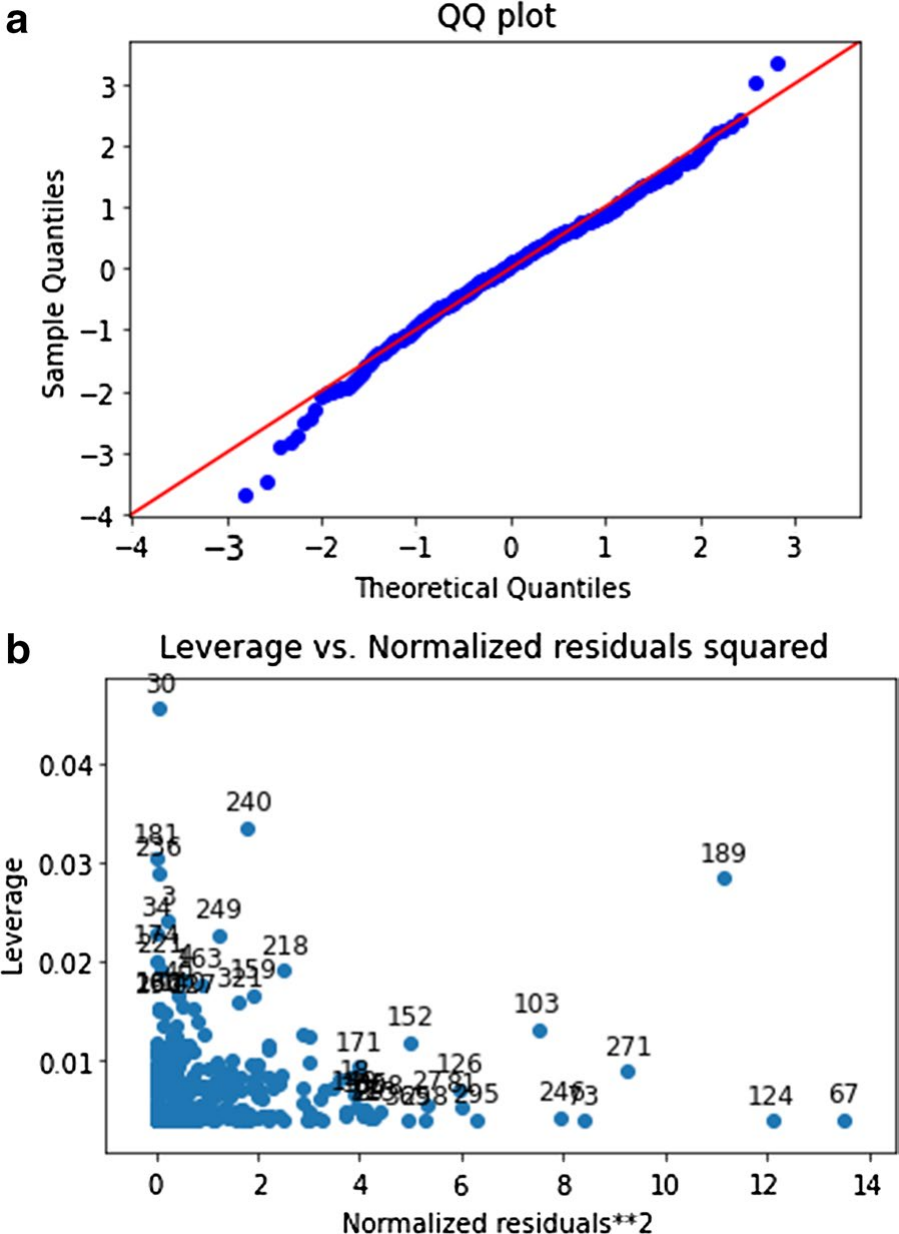
Robustness indicators of the proposed new prediction formula: QQ plot, displaying stringent linearity for theoretical (X-axis) over sample (Y-axis) quantiles (**a**) and leverage vs. normalized residuals squared plot, showing–as desired–significant clustering of values around the origin of the graph (**b**). Numbers in the figure are the tuple of the leverage of the data point and the normalized squared residual

**Fig. 3:**
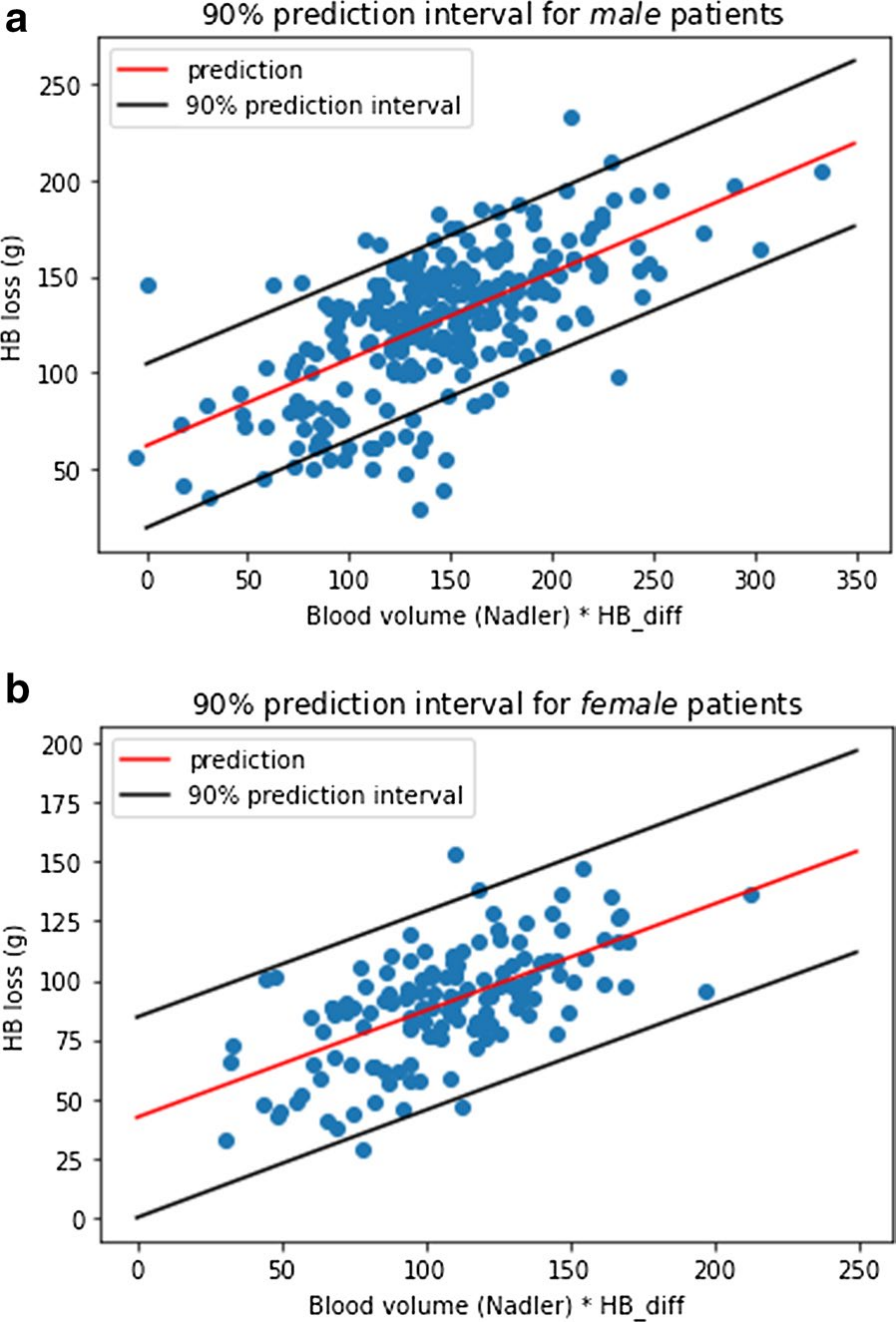
90% Prediction intervals for the heteroscedasticity robust model for the subgroups of male (**a**) and female (**b**) donors. The X-axis shows the product of the blood volume determined via the Nadler formula and the difference in Hb concentration before and after the bloodletting

All coefficients were significant at a < 0.1% significance level. We performed the standard diagnostic tests to ensure the assumptions of a linear regression model are satisfied and the model is robust. First, the condition number of 1.46 indicated that the model does not suffer from severe multicollinearity. Second, the partial regression and partial residual plots confirmed the linear relationship between the variables and hemoglobin loss. Third, a Bruesch-Pagan test found significant evidence of heteroscedasticity (p-value < 0.1%). To this end, we re-performed the regression model using a heteroscedasticity consistent covariance matrix to obtain robust coefficient standard errors. Under this heteroscedasticity robust model, we found that each variable remained significant at a < 0.1% significance level. Fourth, the Q-Q plot (Fig. 2a) indicated that the distribution of the residuals was reasonably close to the normal distribution with only very slightly heavier tails. Nevertheless, the Jarque–Bera test showed that our models violated the normal distribution assumption for the residuals. Last, plotting the leverage against the normalized residuals squares (Fig. 2b) indicated the presence of several outliers with large residuals or large leverage. In order to address the issues of non-normality of the residuals and presence of several outliers, we performed a robust regression with MM estimators. This robust regression yielded coefficients within one standard error of our original model, so we conclude that our original model is reasonably robust against the non-normality of residuals, heteroscedasticity and the presence of outliers. The prediction intervals of the heteroscedasticity robust model are depicted in Fig. 3. The code used to perform the analyses is provided as Additional files 2 and 3.

**Fig. 4:**
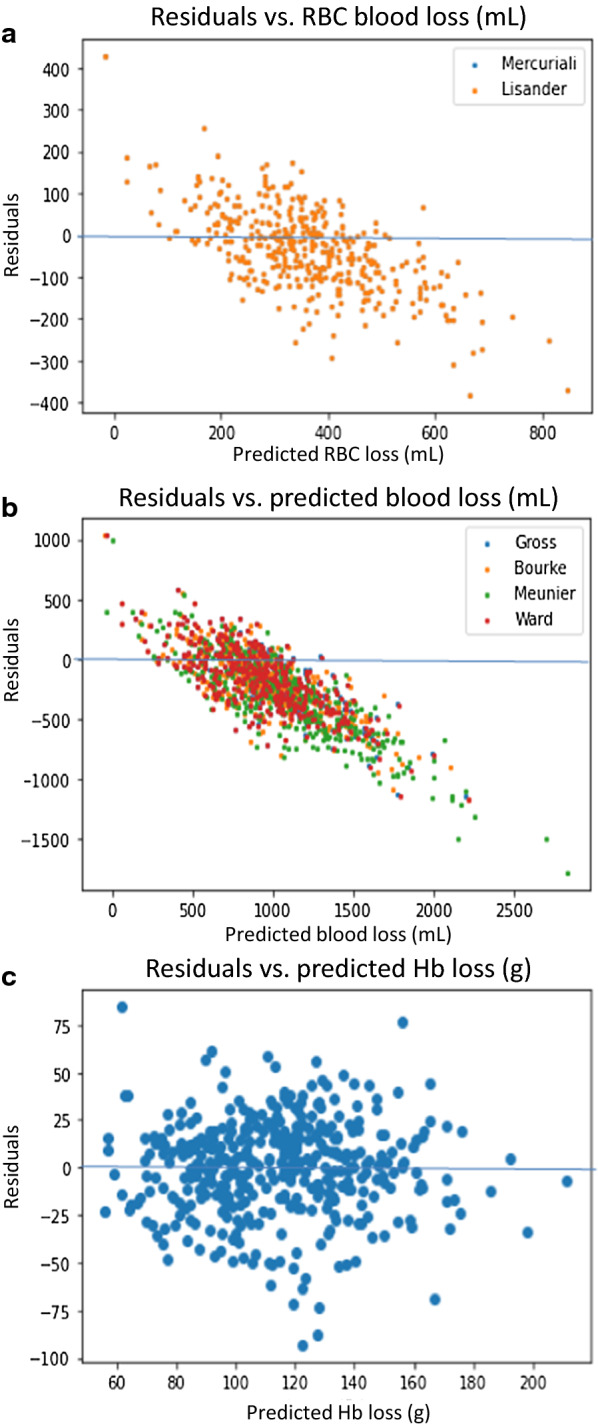
Residuals versus predicted values for the formulas of Mercuriali/ Lisander (same formula, so long as no blood is transfused; RBC loss in mL) (**a**), Gross, Bourne, Meunier and Ward (blood loss in mL) (**b**) and for the new formula presented here (Hb loss in g) (**c**). The significant slope of all formulas in **a** and **b** is apparent, indicating systematic underestimation of small and overestimation of large blood loss, as well as the mean of residuals for all formulas is markedly below zero, i.e. in the mean, blood loss is systematically underestimated by all these formulas. By contrast, the mean value of residuals and the gradient with the new formula (**c**) are zero, i.e. the predicted Hb loss is equally precise and accurate throughout the entire range of observations

Several blood or hemoglobin loss estimates have been proposed [16–22], their development footing on reasonable expectations, i.e. that blood volume would rapidly re-equilibrate, causing Hb dilution which would be reflective of Hb loss. The majority of these formulas were either not validated in individuals with volume-controlled bleeds, validated for one volume [17], or in a very small number of patients and targeting an Hct representing a very large blood loss, namely 30% [38]. We used our dataset to ascertain the quality of these alternative prediction algorithms, paying specific attention to the quality of the predictions at the extremes, i.e. small and large donors, small and large hemoglobin predicted loss, as well as linearity. Of note, our formula is not hypothesis-driven but generated using an unbiased “big data” approach where the use of any readily available observed values is permitted. The correlation coefficients of predicted and observed blood loss for the individual formulas are displayed in Table 2. Figure 4 features the plots of residual (actual versus predicted loss) against predicted loss. As is apparent, at the Hb dilution caused by a blood loss of 500 mL, all formulas calculate approximately the correct blood loss volume or the equivalent loss of Hb mass or RBC volume (residuals of approximately zero). Across all volumes, however, the fit of all the formulas is modest. In particular, depending on the model the average prediction is 10–30% below the actual loss. Moreover, all formulas systematically underestimate blood loss in low-prediction and overestimate blood loss in high-prediction intervals.

**Table 2:** Correlation coefficient and precision of prediction formulas

Model	Prediction	Correlation coefficient	Hb equivalent mean absolute error (MAE)
Our formula	HB loss (g)	72.9%	19.6
Mercuriali	RBC loss (mL)	67.7%	20.8
Lisander	RBC loss (mL)	67.7%	20.8
Bourke	Blood loss (mL)	60.5%	20.0
Ward	Blood loss (mL)	58.9%	20.5
Gross	Blood loss (mL)	59.0%	20.5
Meunier	Blood loss (mL)	57.5%	21.0

The novel formula as well as existent prediction formulas were validated against the controlled blood loss cohort, each for the parameter they are supposed to predict. The novel formula is characterized by the highest correlation coefficient and smallest mean absolute error (Hb equivalent MAE; 1 g of Hb ≈ 2.87 mL RBC ≈ 6.79 mL blood loss), i.e. is markedly more accurate than available formulas with approximately equal precision

## Discussion

The ability to determine intra-operative blood, RBC or hemoglobin loss is a highly meaningful measure for many quality management exercises, whether for comparison of surgical techniques [1–12], of different hospitals or of individual surgeons. For such exercises, linearity is the most important criterion, so that twice as high actual blood loss is reflected by twice as high a calculated value, importantly also at smaller blood volumes which do not change Hb much. “Linearity” is here used not to describe the mathematical transaction of observed values, but instead to indicate the correlation between observed and calculated blood loss. The existing formulas on average correctly calculate a blood loss of 500 mL as 500 mL, but a reduction of the blood loss by half (to 250 mL) is predicted as no blood loss at all. Using these formulas to calculate blood loss associated with a novel surgical technique will thus exaggerate the improvement brought on by the new technique. A formula which in addition to being linear is also accurate can serve to generate benchmarking data for certain surgical interventions, particularly if the precision is also quite good. For patients with reduced compensatory capacity, such data could then support decision making with respect to ordering of transfusion products and thus improve inventory management and reduce costs.

Surgeon- or anesthesist-estimated blood loss is highly imprecise, highly subjective and systematically much too low, i.e. of modest usefulness [13]. For many decades this problem has been recognized, and a number of more objective solutions have been proposed, namely simple mathematical formulas using pre- and post-surgical measures of hemoglobin or hematocrit [16–22]. Although the laboratory values needed for their application are readily available, none of the formulas have caught on, indicating their limited usefulness. And indeed as we are showing by comparing predicted and observed blood loss based on our controlled blood loss cohort, none of the formulas make predictions which are linear across a relevant range, let alone accurate or precise, as was expected based on their mathematical simplicity.

We therefore present a novel formula for hemoglobin loss calculation which was generated using modern mathematical tools. The formula is based on and was validated against a cohort of > 400 individuals subjected to controlled bloodletting across volumes equivalent to between 0.5 and 4.5 RBC units. The formula incorporates sex, height, weight and hemoglobin concentration in blood immediately before and 24–28 h after the controlled bloodletting. At a first glance rather surprisingly, of the different mathematical approaches applied, the fairly simple regression models performed much better than more advanced techniques such as neural networks, an observation we attribute to the limited size of the dataset. The precision of the novel formula is such that across the entire range of volumes–the equivalent of 29 to 233 g of hemoglobin–the hemoglobin loss was predicted with an average absolute error of 19.6 g (Table 2), the correlation between predicted and observed hemoglobin loss was strictly linear and the mean difference between predicted and observed hemoglobin loss for all donors was zero throughout the entire range of observed values. The accuracy of the determination is also not affected by donor body mass (not shown), likely due to the relatively modest effect of body mass on blood volume [39] compared to the modest precision of our formula. Whether blood loss in mL, RBC loss in mL, or hemoglobin loss in g is predicted is relatively immaterial due to the relatively tight correlation of the values and the ease with which they can be converted. Since the parameter primarily determining the need for “blood transfusion” treatment (together with the clinical picture) is hemoglobin, and since the remedy for severe anemia, packed red cells, comes in units of 50 g hemoglobin, we opted to generate a formula predicting hemoglobin loss. Pre-surgical Hb concentration can be used to translate hemoglobin loss to blood loss, multiplication of Hb loss with 2.87 roughly translates it to lost RBC volume except in patients with relevant micro- or macrocytic anemia.

While Hb loss from intra- and extravascular Hb stores during a bone marrow harvest can be very precisely ascertained, whether it appropriately models surgical blood loss is not self-evident. Several pieces of evidence suggest, however, that it is. First, where marrow (extravascular blood) is removed, it is replaced by blood. Second, if we look at Fig. 4b we see that the Meunier formula [17] which was developed and validated on the 500 mL blood loss of a blood donation had zero residuals at the 500 ml marrow aspiration volume. Thus for the one volume where data are available the effect of controlled bloodletting and marrow aspiration on Hb dilution is the same.

The price for the improved precision and linearity of the hemoglobin loss calculator is a formula too complex for mental arithmetic. To facilitate use of the formula in the clinic, we therefore generated a worksheet into which the relevant variables are entered and the predicted median and 90% range are calculated. The worksheet is included with the paper as Additional files 1, 2 and 3. For not Hb-relevant blood loss the formula will predict a range for Hb loss which includes zero. This is in consequence of the fact that significant blood loss can occur before Hb will begin to drop, but obviously unchanged Hb may also indicate the absence of a blood loss. Accordingly, for very minor blood loss as occurs during smaller surgical procedures like tonsillectomy [40] our tool is not going to be useful. The precision of the prediction being similar for all prediction algorithms is unsurprising since all are based on pre- and post-surgical Hb concentration.

Limitations of our study include that the formula will not be useful for estimation of minor, not Hb-relevant hemorrhage, and it was not validated for individuals with pre-existing anemia or not normovolemic patients, such as pregnant women. Minor Hb loss into connective tissue overlying the spina iliaca posterior superior from where bone marrow is aspirated will remain unaccounted for but is unlikely to relevantly affect the outcome of the analyses. Although the very refined study of Meunier et al.[17] teaches that normovolemia is not yet achieved within one day even after an incurred blood loss of only half a liter, a single blood draw after 24 h guided the development of our algorithm. It is possible that the timing of this blood draw is responsible for the modest precision of our algorithm. We did not test whether a blood draw immediately after the end of the procedure or at some much later time point could have improved prediction. We believe, however, that the morning-after blood draw is most easily integrated into clinical routines.

In summary, we have developed a calculator for estimation of surgical hemoglobin loss. Its strict linearity, high accuracy (mean error being zero) and reasonable precision predispose its use for intra- and inter-hospital benchmarking of surgical procedures and blood inventory management. Until the algorithm has been validated in an independent cohort it should not be used for medical decision-making.

## Supplementary Information

**Additional file 1.** Excel worksheet for volume loss calculation. Values are pasted into the respective fields, ENTER calculates the incurred blood loss (mean, range).

**Additional file 2.** Model Selection

**Additional file 3.** Linear Regression

## Data Availability

The datasets analysed during the current study are available from the corresponding author on reasonable request.

## Abbreviations

API: Application programming interface
BMI: Body mass index
CBC: Complete blood count
EV: Explained variance
Hb: Hemoglobin
Hct: Hematocrit
IQR: Inter-quartile range
JACIE: Joint Accreditation Committee of the International Society of Cell Therapy ISCT and European Bone Marrow Transplantation Group EBMT
MSE: Mean squared error
Plt: Platelet, thrombocyte
RBC: Red blood cell, erythrocyte
RBF: Radial basis function
ReLu: Rectified linear unit
WMDA: World Marrow Donor Association
