# Supplementary material for: Generation and validation of a formula to calculate hemoglobin loss on a cohort of healthy adults subjected to controlled blood loss
Source: J Transl Med. 2021 Mar 20;19:116. doi: 10.1186/s12967-021-02783-9 (PMC7981850; doi:10.1186/s12967-021-02783-9)
Supplement: Supplementary file 2 — Additional file 2. Model Selection [file 12967_2021_2783_MOESM2_ESM.pdf]

Blood loss prediction - Model selection

Import

In [57]:
```
import pandas as pd
import numpy as np

from sklearn.model_selection import train_test_split, KFold, validation_
curve, learning_curve, GridSearchCV
from sklearn.preprocessing import StandardScaler
from sklearn.metrics import mean_squared_error, mean_absolute_error, r2_
score, explained_variance_score
from sklearn.linear_model import LinearRegression, Ridge
from sklearn.tree import DecisionTreeRegressor
from sklearn.ensemble import AdaBoostRegressor, RandomForestRegressor
from sklearn.svm import SVR
from sklearn.neighbors import KNeighborsRegressor

import matplotlib.pyplot as plt
import seaborn as sn

import warnings
warnings.filterwarnings("ignore")
```

Preprocessing

Data import

In [58]:
```
raw_data = pd.read_excel('Blood_loss_raw_data.xlsx')
raw_data['BMI'] = raw_data['weight']/((raw_data['height']/100)**2)
raw_data.drop(['VU_Eisen','VU_Ferritin', 'loss_eryl'], inplace=True, ax
is=1)
raw_data.dropna(inplace=True)
raw_data.reset_index(inplace=True)
raw_data
```

Out[58]:

	index	weight	sex	height	age	HB_pre	HB_post	HCT_pre	HCT_post	BBS1PLT
0	0	143.0	1	184	30	16.8	15.0	46.4	41.9	199
1	1	88.0	1	185	38	14.5	12.2	40.6	34.4	257
2	2	75.0	0	175	40	14.4	12.7	40.9	35.7	276
3	3	105.0	1	186	44	16.0	13.7	45.6	39.2	243
4	4	88.0	0	178	33	12.8	12.2	36.6	34.6	214
...	...	...	...	...	...	...	...	...	...	...
394	397	86.0	1	190	40	14.0	11.0	43.6	33.8	293

395	398	93.0	1	180	56	15.6	12.0	43.9	34.0	231
396	399	102.0	1	181	24	16.2	12.5	46.4	35.6	271
397	400	87.0	1	189	24	15.0	11.1	41.6	30.7	286
398	401	95.0	1	180	25	14.8	14.8	41.4	41.7	231

399 rows × 18 columns

Data transformation & visualizations

In [62]:
```
# decorrelation of variables
raw_data_temp = raw_data[['weight', 'age', 'height', 'HB_pre', 'HB_post
', 'HCT_pre', 'HCT_post', 'BBS1PLT', 'BBS1LEUCO', 'loss_g']]
raw_data_temp = raw_data_temp.rename(columns={"BBS1PLT": "PLT_pre", 'BB
S1LEUCO': 'LEUCO_pre', 'loss_g': 'HB_loss'})
cm = raw_data_temp.corr().round(2)
sn.heatmap(cm, annot=False, cmap='Reds',fmt='.2g')
plt.show()
```

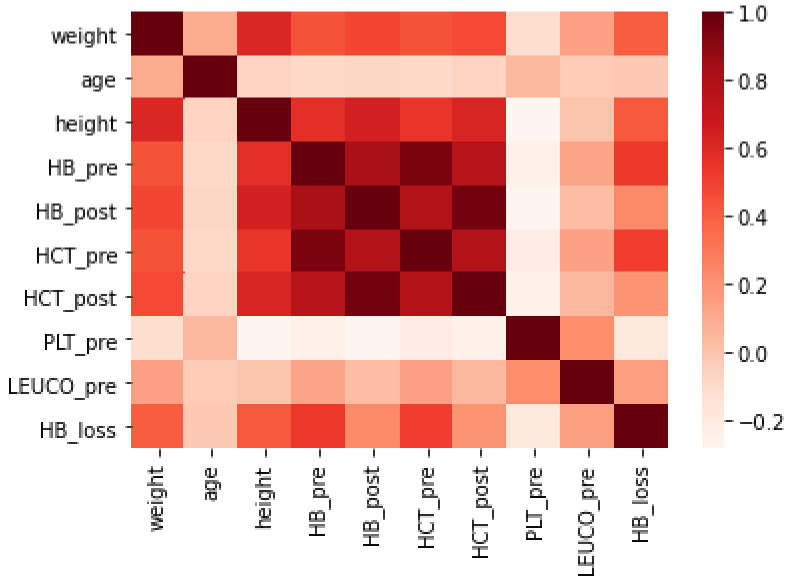

In [11]:
```
print('Quotient of HCT and HB: {}'.format((sum(raw_data['HCT_pre'])+sum
(raw_data['HCT_post']))/(sum(raw_data['HB_pre'])+sum(raw_data['HB_post'
]))))
```

```
Quotient of HCT and HB: 2.870124945665747
```

In [12]:
```
# replace HB_post by HB_diff
raw_data['HB_diff'] = raw_data['HB_post'] - raw_data['HB_pre']
raw_data.drop('HB_post', inplace=True, axis=1)

# correct HCT by HB and calculate difference
raw_data['HCT_diff'] = (raw_data['HCT_post'] - raw_data['HCT_pre'])/2.8
7 - raw_data['HB_diff']
raw_data['HCT_pre'] = raw_data['HCT_pre']/2.87 - raw_data['HB_pre']
raw_data.drop('HCT_post', inplace=True, axis=1)

# dropt BBS4PLT and BBS4LEUCO2 by difference
raw_data.drop(['BBS4PLT', 'BBS4LEUCO2'], inplace=True, axis=1)
```

In [13]:
```
# check correlation matrix
raw_data_temp = raw_data[['HB_pre', 'HB_diff', 'HCT_pre', 'HCT_diff', 'BBS1PLT', 'BBS1LEUCO']]
cm = raw_data_temp.corr()
sn.heatmap(cm, annot=True, cmap='Reds', fmt='.2g')
plt.show()
```

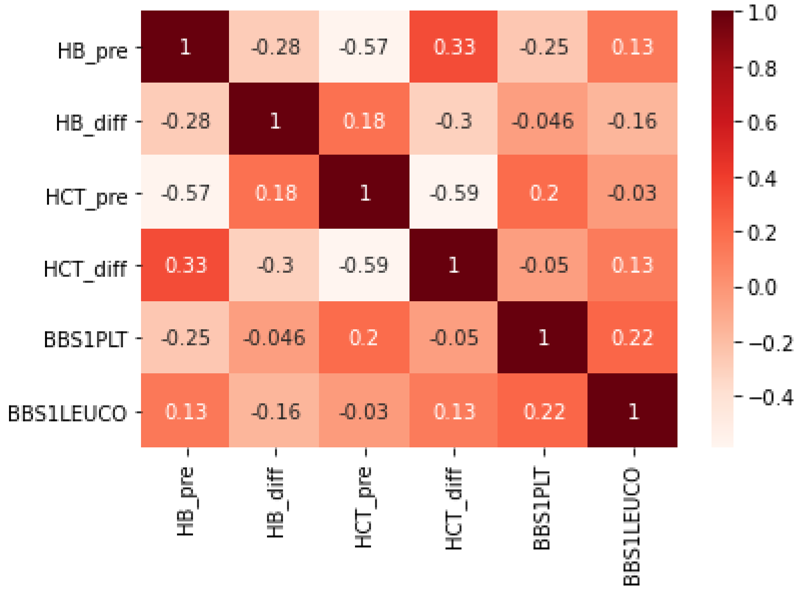

In [14]:
```
# weight
raw_data['weight'].plot.hist(grid=True, bins=20, rwidth=0.9, color='#607c8e')
plt.title('Distribution of weight')
# to be log transformed
```

Out[14]: `Text(0.5, 1.0, 'Distribution of weight')`

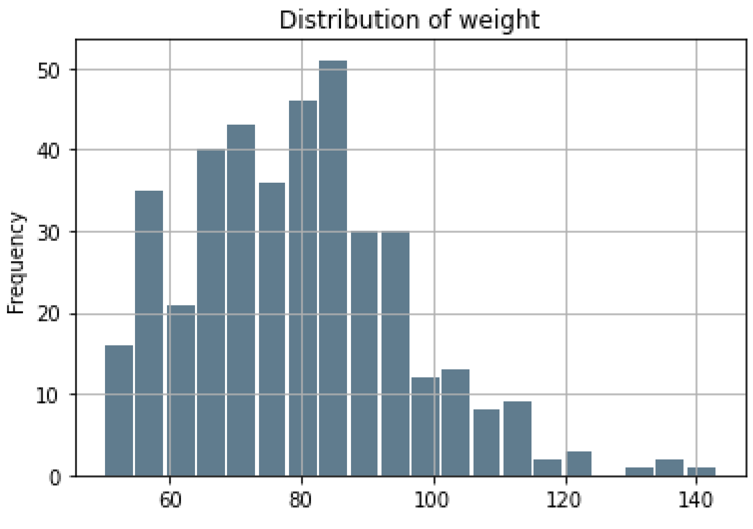

In [15]:
```
# height
raw_data['height'].plot.hist(grid=True, bins=20, rwidth=0.9, color='#607c8e')
plt.title('Distribution of height')
```

Out[15]: `Text(0.5, 1.0, 'Distribution of height')`

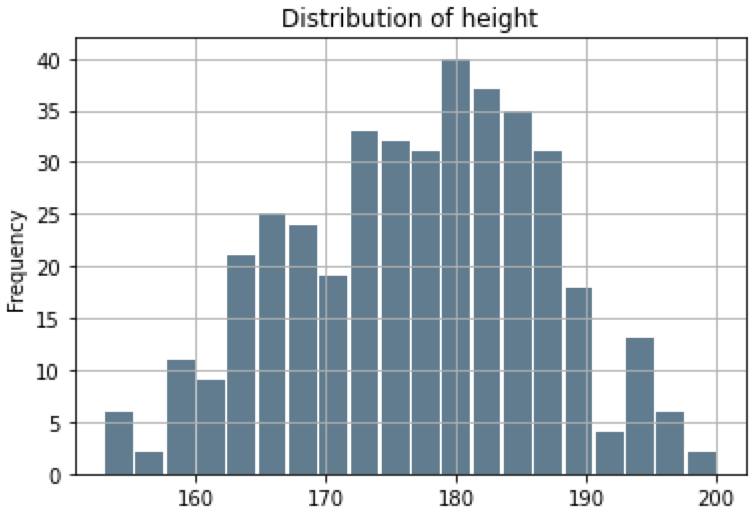

In [16]:
```
# BMI
raw_data['BMI'].plot.hist(grid=True, bins=20, rwidth=0.9, color='#607c8
e')
plt.title('Distribution of BMI')
# to be log transformed
```

Out[16]: Text(0.5, 1.0, 'Distribution of BMI')

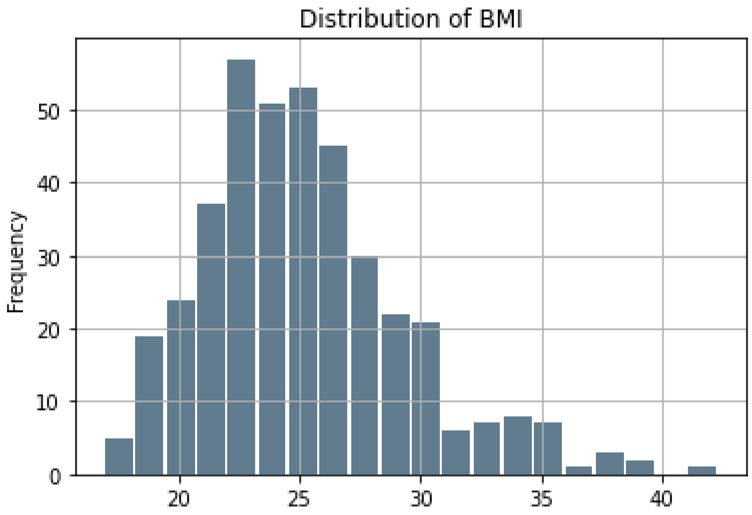

In [17]:
```
# age
raw_data['age'].plot.hist(grid=True, bins=20, rwidth=0.9, color='#607c8
e')
plt.title('Distribution of age')
# to be log transformed
```

Out[17]: Text(0.5, 1.0, 'Distribution of age')

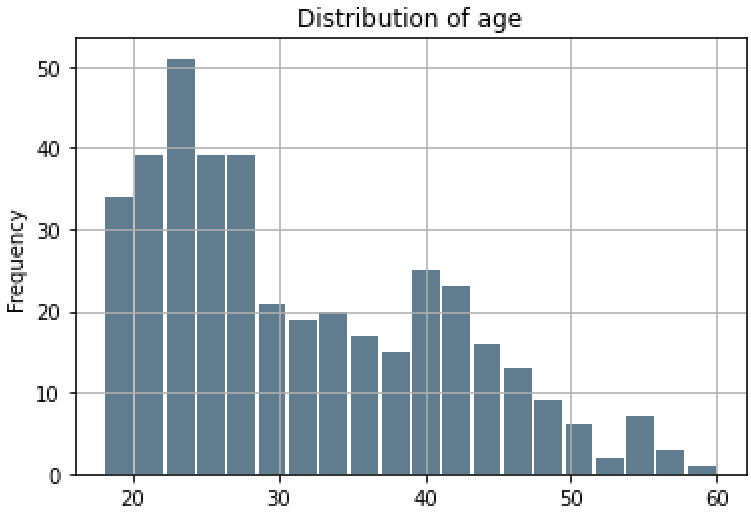

In [18]:
```
# HB_pre
raw_data['HB_pre'].plot.hist(grid=True, bins=20, rwidth=0.9, color='#60
7c8e')
plt.title('Distribution of HB_pre')
```

Out[18]: Text(0.5, 1.0, 'Distribution of HB_pre')

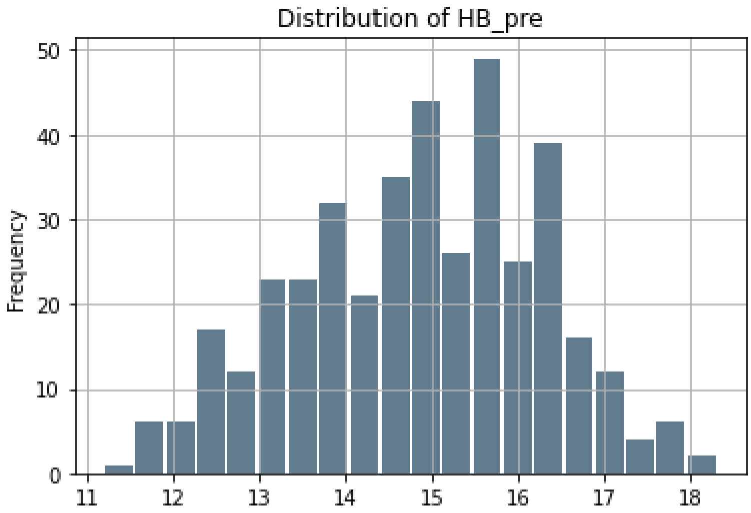

In [19]:
```
# HB_diff
raw_data['HB_diff'].plot.hist(grid=True, bins=20, rwidth=0.9, color='#6
07c8e')
plt.title('Distribution of HB_diff')
```

Out[19]: Text(0.5, 1.0, 'Distribution of HB_diff')

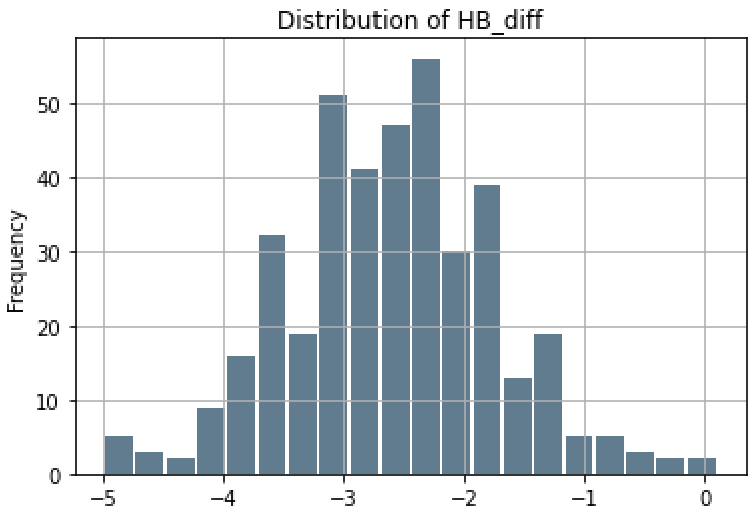

In [20]:
```
# HCT_pre
raw_data['HCT_pre'].plot.hist(grid=True, bins=20, rwidth=0.9, color='#607c8e')
plt.title('Distribution of HCT_pre')
```

Out[20]: Text(0.5, 1.0, 'Distribution of HCT_pre')

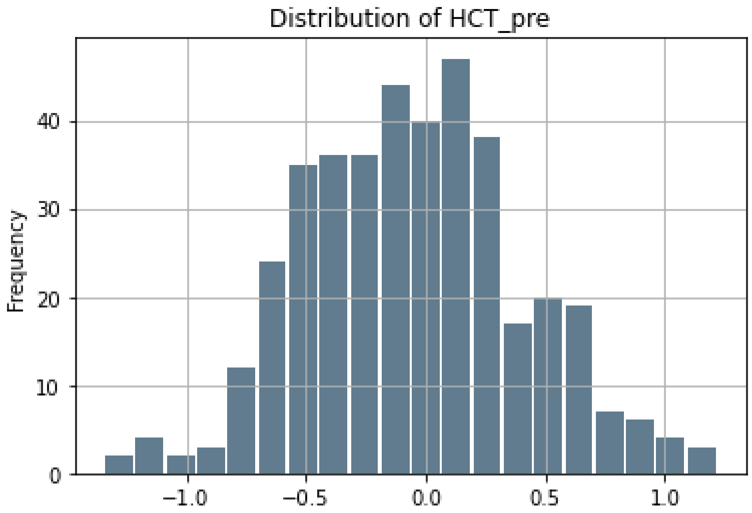

In [21]:
```
# HCT_diff
raw_data['HCT_diff'].plot.hist(grid=True, bins=20, rwidth=0.9, color='#607c8e')
plt.title('Distribution of HCT_diff')
```

Out[21]: Text(0.5, 1.0, 'Distribution of HCT_diff')

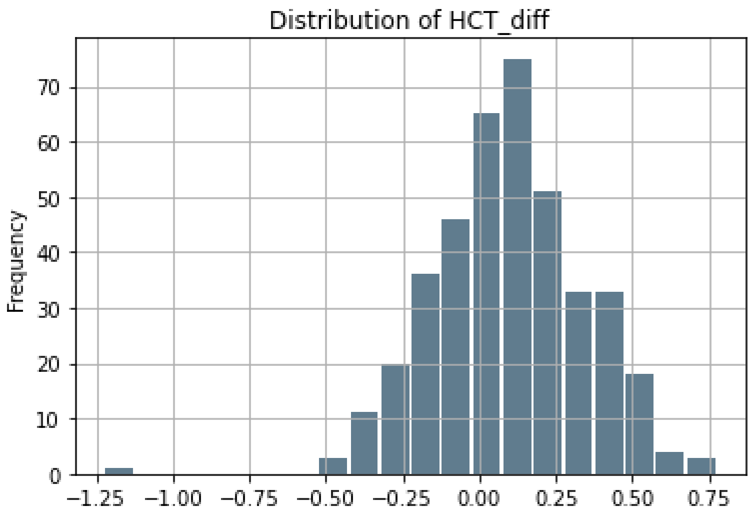

In [22]:
```
# BBS1PLT
raw_data['BBS1PLT'].plot.hist(grid=True, bins=20, rwidth=0.9, color='#6
07c8e')
plt.title('Distribution of BBS1PLT')
```

Out[22]: Text(0.5, 1.0, 'Distribution of BBS1PLT')

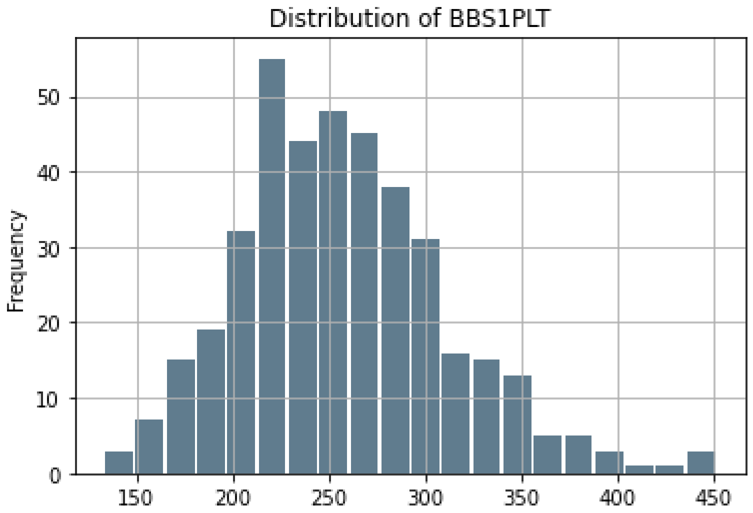

In [23]:
```
# BBS1LEUCO
raw_data['BBS1LEUCO'].plot.hist(grid=True, bins=20, rwidth=0.9, color='
#607c8e')
plt.title('Distribution of BBS1LEUCO')
# to be log-transformed
```

Out[23]: Text(0.5, 1.0, 'Distribution of BBS1LEUCO')

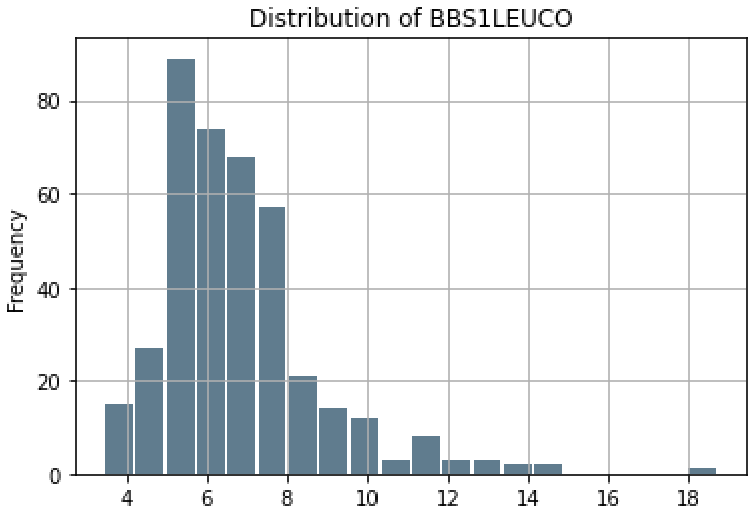

In [24]:
```
# Nadler
raw_data['Nadler'].plot.hist(grid=True, bins=20, rwidth=0.9, color='#607c8e')
plt.title('Distribution of Nadler')
```

Out[24]: Text(0.5, 1.0, 'Distribution of Nadler')

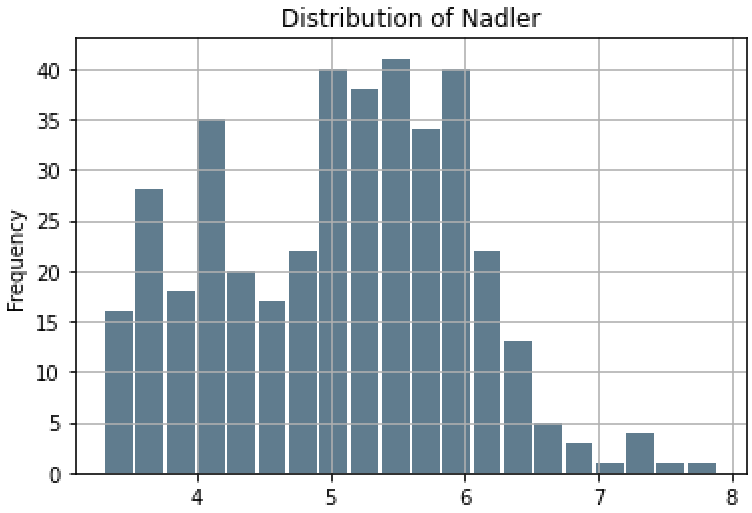

In [25]:
```
# Nadler * HB_diff
raw_data['BV_HB'].plot.hist(grid=True, bins=20, rwidth=0.9, color='#607c8e')
plt.title('Distribution of BV_HB')
```

Out[25]: Text(0.5, 1.0, 'Distribution of BV_HB')

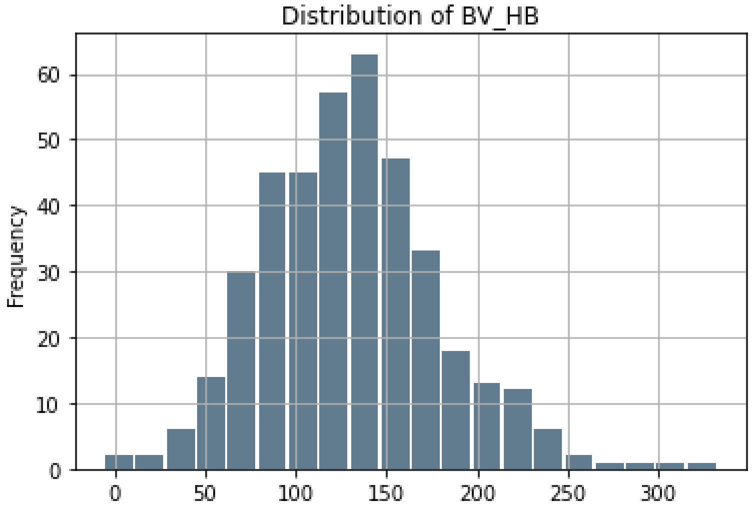

In [26]:
```
# blood loss (g)
raw_data['loss_g'].plot.hist(grid=True, bins=20, rwidth=0.9, color='#60
7c8e')
plt.title('Distribution of blood_loss (g)')
```

Out[26]:
```
Text(0.5, 1.0, 'Distribution of blood_loss (g)')
```

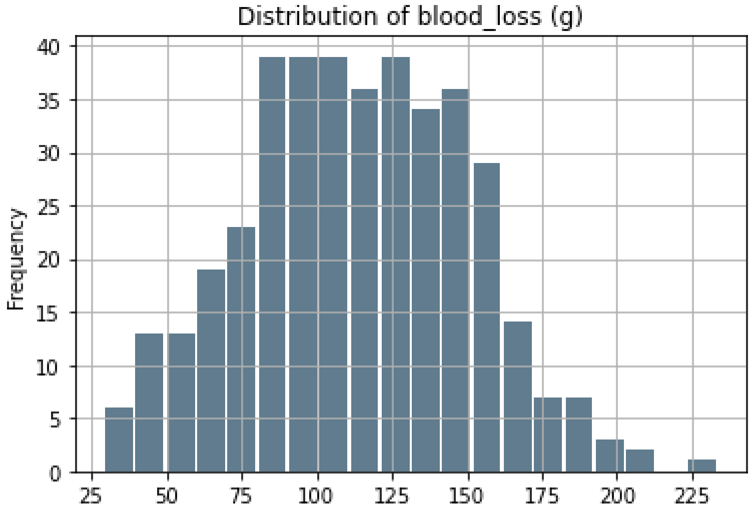

In [27]:
```
# blood loss (ml)
raw_data['loss_ml'].plot.hist(grid=True, bins=20, rwidth=0.9, color='#6
07c8e')
plt.title('Distribution of blood_loss (ml)')
```

Out[27]:
```
Text(0.5, 1.0, 'Distribution of blood_loss (ml)')
```

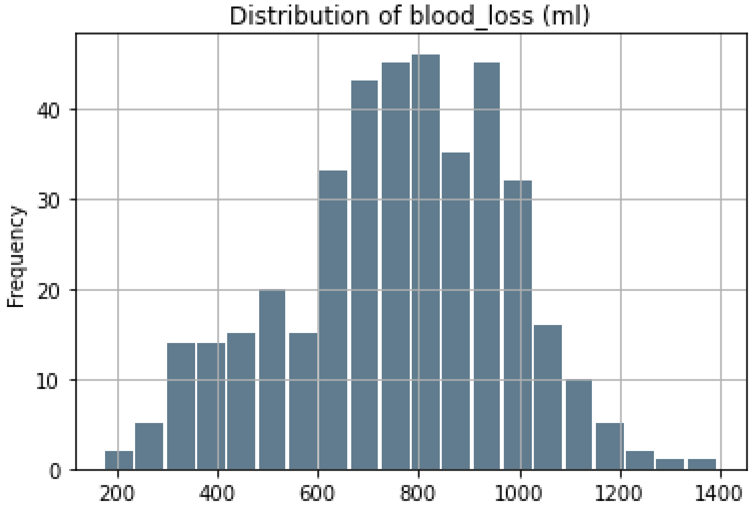

Data transformations

In [28]:
```
# log transformations
raw_data['weight'] = np.log(raw_data['weight'])
raw_data['age'] = np.log(raw_data['age'])
raw_data['BMI'] = np.log(raw_data['BMI'])
raw_data['BBS1LEUCO'] = np.log(raw_data['BBS1LEUCO'])

# split into training and testing set
y = raw_data['loss_g']
X = raw_data.drop(['loss_g', 'loss_ml'], axis=1)
X_train_all, X_test_all, y_train, y_test = train_test_split(X, y, test_
size=0.25, random_state=1)

X_train_med = pd.DataFrame(X_train_all['BV_HB'])
X_test_med = pd.DataFrame(X_test_all['BV_HB'])
X_train = X_train_all[['weight', 'sex', 'height', 'BMI', 'age', 'HB_pre
', 'HCT_pre', 'HCT_diff', 'BBS1PLT', 'BBS1LEUCO', 'BV_HB']]
X_test = X_test_all[['weight', 'sex', 'height', 'BMI', 'age', 'HB_pre',
'HCT_pre', 'HCT_diff', 'BBS1PLT', 'BBS1LEUCO', 'BV_HB']]
X_all = X[['weight', 'sex', 'height', 'BMI', 'age', 'HB_pre', 'HCT_pre'
, 'HCT_diff', 'BBS1PLT', 'BBS1LEUCO', 'BV_HB']]
X_med = pd.DataFrame(X['BV_HB'])

# standard scaling
scaler = StandardScaler()
X_train[['weight', 'height', 'BMI', 'age', 'HB_pre', 'HCT_diff', 'BBS1P
LT', 'BBS1LEUCO', 'BV_HB']] = scaler.fit_transform(X_train[['weight', '
height', 'BMI', 'age', 'HB_pre', 'HCT_diff', 'BBS1PLT', 'BBS1LEUCO', 'B
V_HB']])
X_test[['weight', 'height', 'BMI', 'age', 'HB_pre', 'HCT_diff', 'BBS1PL
T', 'BBS1LEUCO', 'BV_HB']] = scaler.transform(X_test[['weight', 'height
', 'BMI', 'age', 'HB_pre', 'HCT_diff', 'BBS1PLT', 'BBS1LEUCO', 'BV_HB']
])
```

Medical Model

In [60]:
```
from sklearn.linear_model import LinearRegression

reg = LinearRegression().fit(X_train_med, y_train)
y_pred = reg.predict(X_test_med)
```

```
MSE_reg = mean_squared_error(y_test, y_pred)
EV_reg = explained_variance_score(y_test, y_pred)

print('Medical Model: MSE: {}, EV: {}'.format(MSE_reg, EV_reg))
print('R^2 value:{}'.format(reg.score(X_train_med, y_train)))
```

```
Medical Model: MSE: 696.0207038872849, EV: 0.5419639699004295
R^2 value:0.455254022565739
```

In [30]:

```
import statsmodels.api as sm
from scipy import stats

X_med2 = sm.add_constant(X_med)
est = sm.OLS(y, X_med2).fit()
print(est.summary())
```

```
                            OLS Regression Results

==============================================================================
======
Dep. Variable:                 loss_g   R-squared:                       0
.476
Model:                            OLS   Adj. R-squared:                  0
.474
Method:                 Least Squares   F-statistic:                     3
60.3
Date:                Tue, 29 Sep 2020   Prob (F-statistic):           1.2
2e-57
Time:                        11:13:00   Log-Likelihood:                -18
77.3
No. Observations:                 399   AIC:                             3
759.
Df Residuals:                     397   BIC:                             37
67.
Df Model:                           1

Covariance Type:            nonrobust

==============================================================================
======
                coef    std err          t      P>|t|      [0.025      0.
975]
------------------------------------------------------------------------------
----------
const        46.0209      3.808     12.087      0.000      38.536      53
.506
BV_HB         0.5167      0.027     18.982      0.000       0.463       0
.570
==============================================================================
======
Omnibus:                        2.558   Durbin-Watson:                   1
.058
Prob(Omnibus):                  0.278   Jarque-Bera (JB):                2
.625
Skew:                           0.055   Prob(JB):                        0.
269
Kurtosis:                       3.382   Cond. No.
397.
==============================================================================
======
```

```
Warnings:
[1] Standard Errors assume that the covariance matrix of the errors is c
orrectly specified.
```

Linear Regression

In [33]:
```
# hyperparameter tuning
X_train_lin = X_train[['sex', 'BV_HB']]
X_test_lin = X_test[['sex', 'BV_HB']]
```

In [34]:
```
reg2 = LinearRegression().fit(X_train_lin, y_train)
y_pred = reg2.predict(X_test_lin)

MSE_reg2 = mean_squared_error(y_test, y_pred)
EV_reg2 = explained_variance_score(y_test, y_pred)

print('Linear Regression: MSE: {}, EV: {}'.format(MSE_reg2, EV_reg2))
```

```
Linear Regression: MSE: 614.2440384546053, EV: 0.601400547271544
```

In [35]:
```
X_all
```

Out[35]:

	weight	sex	height	BMI	age	HB_pre	HCT_pre	HCT_diff	BBS1PLT	BBS
0	4.962845	1	184	3.743313	3.401197	16.8	-0.632753	0.232056	199	
1	4.477337	1	185	3.246966	3.637586	14.5	-0.353659	0.139721	257	
2	4.317488	0	175	3.198257	3.688879	14.4	-0.149129	-0.111847	276	
3	4.653960	1	186	3.412807	3.784190	16.0	-0.111498	0.070035	243	
4	4.477337	0	178	3.324110	3.496508	12.8	-0.047387	-0.096864	214	
...	...	...	...	...	...	...	...	...	...	
394	4.454347	1	190	3.170640	3.688879	14.0	1.191638	-0.414634	293	
395	4.532599	1	180	3.357026	4.025352	15.6	-0.303833	0.150523	231	
396	4.624973	1	181	3.438319	3.178054	16.2	-0.032753	-0.063066	271	
397	4.465908	1	189	3.192754	3.178054	15.0	-0.505226	0.102091	286	
398	4.553877	1	180	3.378304	3.218876	14.8	-0.374913	0.104530	231	

399 rows × 11 columns

In [39]:
```
import statsmodels.api as sm
from scipy import stats

X_all2 = X_all[['sex', 'BV_HB']]
#X_all2['sex_BV_HB'] = X_all2['sex'] * X_all2['BV_HB']
X_all2 = sm.add_constant(X_all2)
est = sm.OLS(y, X_all2).fit()
print(est.summary())
```

```
                            OLS Regression Results
==============================================================================
```

```
======
Dep. Variable:                 loss_g   R-squared:                       0
.532
Model:                            OLS   Adj. R-squared:                  0
.529
Method:                 Least Squares   F-statistic:                     2
24.8
Date:                Tue, 29 Sep 2020   Prob (F-statistic):           5.8
1e-66
Time:                        11:14:44   Log-Likelihood:                -18
54.8
No. Observations:                 399   AIC:                             3
716.
Df Residuals:                     396   BIC:                            37
28.
Df Model:                           2

Covariance Type:            nonrobust

========================================================================
======
                 coef    std err          t      P>|t|      [0.025      0.
975]
------------------------------------------------------------------------
----------
const         42.5130      3.639     11.682      0.000      35.358      49
.668
sex           19.5454      2.842      6.877      0.000      13.957      25
.133
BV_HB          0.4477      0.028     16.194      0.000       0.393       0
.502
========================================================================
======
Omnibus:                       13.772   Durbin-Watson:                   0
.979
Prob(Omnibus):                  0.001   Jarque-Bera (JB):               17
.980
Skew:                          -0.313   Prob(JB):                     0.00
0125
Kurtosis:                       3.830   Cond. No.
407.
========================================================================
======

Warnings:
[1] Standard Errors assume that the covariance matrix of the errors is c
orrectly specified.
```

Ridge Regression

In [40]:

```
# cross-validated grid search
parameters = {'alpha': np.arange(1,20)}
reg3_base = Ridge(random_state=0)
reg3 = GridSearchCV(reg3_base, parameters, scoring='neg_mean_squared_err
or', cv=5).fit(X_train, y_train)
```

In [41]:

```
reg3.best_params_
```

Out[41]:

```
{'alpha': 5}
```

In [42]:
```
y_pred = reg3.predict(X_test)

MSE_reg3 = mean_squared_error(y_test, y_pred)
EV_reg3 = explained_variance_score(y_test, y_pred)

print('Ridge Regression: MSE: {}, EV: {}'.format(MSE_reg3, EV_reg3))
```

```
Ridge Regression: MSE: 612.7910376259383, EV: 0.5954568274744507
```

AdaBoost

In [43]:
```
# grid search with GINI
parameters = {'n_estimators': [50, 75, 100, 125, 150]}
ada_base = AdaBoostRegressor(base_estimator=DecisionTreeRegressor(criter
ion='mse', max_depth=5, ccp_alpha=0.1), random_state=0)
ada = GridSearchCV(ada_base, parameters, scoring='neg_mean_squared_error
', cv=5).fit(X_train, y_train)
```

In [44]:
```
ada.best_params_
```

Out[44]:
```
{'n_estimators': 100}
```

In [45]:
```
y_pred = ada.predict(X_test)

MSE_ada = mean_squared_error(y_test, y_pred)
EV_ada = explained_variance_score(y_test, y_pred)

print('AdaBoostRegressor: MSE: {}, EV: {}'.format(MSE_ada, EV_ada))
```

```
AdaBoostRegressor: MSE: 807.575036440719, EV: 0.4655880194014991
```

Random Forest

In [46]:
```
parameters = {'n_estimators': [30,40,50,60], 'max_depth': [4,5,6,7]}
rf_base = RandomForestRegressor(random_state=0)
rf = GridSearchCV(rf_base, parameters, scoring='neg_mean_squared_error',
cv=5).fit(X_train, y_train)
```

In [47]:
```
rf.best_params_
```

Out[47]:
```
{'max_depth': 4, 'n_estimators': 40}
```

In [48]:
```
y_pred = rf.predict(X_test)

MSE_rf = mean_squared_error(y_test, y_pred)
EV_rf = explained_variance_score(y_test, y_pred)

print('RandomForestRegressor: MSE: {}, EV: {}'.format(MSE_rf, EV_rf))
```

```
RandomForestRegressor: MSE: 726.3067320732474, EV: 0.5122053865071159
```

Support Vector Regression

In [49]:
```
parameters = {'C': np.arange(5,10), 'epsilon': np.arange(0,10)/10}
svr_base = SVR()
svr = GridSearchCV(svr_base, parameters, scoring='neg_mean_squared_error
', cv=5).fit(X_train, y_train)
```

In [50]:
```
svr.best_params_
```

Out[50]:
```
{'C': 9, 'epsilon': 0.9}
```

In [51]:
```
y_pred = svr.predict(X_test)

MSE_svr = mean_squared_error(y_test, y_pred)
EV_svr = explained_variance_score(y_test, y_pred)

print('Support Vector Regression: MSE: {}, EV: {}'.format(MSE_svr, EV_s
vr))
```

```
Support Vector Regression: MSE: 656.8565142629554, EV: 0.548492549075721
2
```

kNN

In [52]:
```
# grid search
parameters = {'n_neighbors': [5,10,20,50, 100]}
knn_base = KNeighborsRegressor()
knn = GridSearchCV(knn_base, parameters, scoring='neg_mean_squared_error
', cv=5, n_jobs=-1).fit(X_train, y_train)
```

In [53]:
```
knn.best_params_
```

Out[53]:
```
{'n_neighbors': 20}
```

In [54]:
```
y_pred = knn.predict(X_test)

MSE_knn = mean_squared_error(y_test, y_pred)
EV_knn = explained_variance_score(y_test, y_pred)

print('kNN regression: MSE: {}, EV: {}'.format(MSE_knn, EV_knn))
```

```
kNN regression: MSE: 682.5534900346695, EV: 0.5365725451450432
```

Neural Network

In [61]:
```
from keras.models import Sequential
from keras.layers.core import Dense, Activation, Flatten, Dropout

# Building the model
model = Sequential()
model.add(Dense(16, input_dim=11, kernel_initializer='normal', activati
on='relu'))
model.add(Dropout(.2))
model.add(Dense(16, activation='relu'))
model.add(Dropout(.2))
model.add(Dense(16, activation='relu'))
model.add(Dropout(.2))
model.add(Dense(1, activation='relu'))
```

```
model.compile(loss='mse', optimizer='adam', metrics=['mse'])

history = model.fit(X_train, y_train, epochs=500, batch_size=25,  verbo
se=1, validation_split=0.2)

y_pred = model.predict(X_test)
MSE_nn = mean_squared_error(y_test, y_pred)
EV_nn = explained_variance_score(y_test, y_pred)

print('Neural Network: MSE: {}, EV: {}'.format(MSE_nn, EV_nn))
```

```
Epoch 1/500
10/10 [==============================] - 0s 20ms/step - loss: 14113.8584
- mse: 14113.8584 - val_loss: 13519.8418 - val_mse: 13519.8418
Epoch 2/500
10/10 [==============================] - 0s 4ms/step - loss: 14094.5752
- mse: 14094.5752 - val_loss: 13496.9121 - val_mse: 13496.9121
Epoch 3/500
10/10 [==============================] - 0s 6ms/step - loss: 14065.7109
- mse: 14065.7109 - val_loss: 13464.9834 - val_mse: 13464.9834
Epoch 4/500
10/10 [==============================] - 0s 4ms/step - loss: 14028.1494
- mse: 14028.1494 - val_loss: 13418.9648 - val_mse: 13418.9639
Epoch 5/500
10/10 [==============================] - 0s 4ms/step - loss: 13970.8936
- mse: 13970.8936 - val_loss: 13353.3662 - val_mse: 13353.3662
Epoch 6/500
10/10 [==============================] - 0s 5ms/step - loss: 13888.2070
- mse: 13888.2070 - val_loss: 13260.9336 - val_mse: 13260.9336
Epoch 7/500
10/10 [==============================] - 0s 5ms/step - loss: 13769.4150
- mse: 13769.4150 - val_loss: 13132.3691 - val_mse: 13132.3691
Epoch 8/500
10/10 [==============================] - 0s 4ms/step - loss: 13604.6953
- mse: 13604.6953 - val_loss: 12953.0615 - val_mse: 12953.0615
Epoch 9/500
10/10 [==============================] - 0s 3ms/step - loss: 13384.6025
- mse: 13384.6025 - val_loss: 12707.0312 - val_mse: 12707.0312
Epoch 10/500
10/10 [==============================] - 0s 4ms/step - loss: 13062.5703
- mse: 13062.5703 - val_loss: 12368.8467 - val_mse: 12368.8467
Epoch 11/500
10/10 [==============================] - 0s 4ms/step - loss: 12627.6797
- mse: 12627.6797 - val_loss: 11922.8184 - val_mse: 11922.8184
Epoch 12/500
10/10 [==============================] - 0s 4ms/step - loss: 12021.8486
- mse: 12021.8486 - val_loss: 11340.2500 - val_mse: 11340.2500
Epoch 13/500
10/10 [==============================] - 0s 3ms/step - loss: 11456.2930
- mse: 11456.2930 - val_loss: 10609.7002 - val_mse: 10609.7012
Epoch 14/500
10/10 [==============================] - 0s 4ms/step - loss: 10672.5527
- mse: 10672.5508 - val_loss: 9720.7500 - val_mse: 9720.7500
Epoch 15/500
10/10 [==============================] - 0s 3ms/step - loss: 9573.9375 -
mse: 9573.9375 - val_loss: 8672.5986 - val_mse: 8672.5986
Epoch 16/500
10/10 [==============================] - 0s 3ms/step - loss: 8268.2861 -
mse: 8268.2861 - val_loss: 7496.7144 - val_mse: 7496.7144
Epoch 17/500
```

```
10/10 [==============================] - 0s 4ms/step - loss: 7063.7998 -
mse: 7063.7998 - val_loss: 6216.3047 - val_mse: 6216.3047
Epoch 18/500
10/10 [==============================] - 0s 3ms/step - loss: 5792.0874 -
mse: 5792.0874 - val_loss: 4962.8462 - val_mse: 4962.8462
Epoch 19/500
10/10 [==============================] - 0s 4ms/step - loss: 4484.7363 -
mse: 4484.7363 - val_loss: 3889.9915 - val_mse: 3889.9915
Epoch 20/500
10/10 [==============================] - 0s 3ms/step - loss: 3625.5305 -
mse: 3625.5305 - val_loss: 3069.6897 - val_mse: 3069.6897
Epoch 21/500
10/10 [==============================] - 0s 3ms/step - loss: 2779.8733 -
mse: 2779.8733 - val_loss: 2502.9844 - val_mse: 2502.9844
Epoch 22/500
10/10 [==============================] - 0s 4ms/step - loss: 2298.4397 -
mse: 2298.4397 - val_loss: 2191.9172 - val_mse: 2191.9172
Epoch 23/500
10/10 [==============================] - 0s 4ms/step - loss: 2386.1108 -
mse: 2386.1108 - val_loss: 2025.3575 - val_mse: 2025.3575
Epoch 24/500
10/10 [==============================] - 0s 4ms/step - loss: 2389.8752 -
mse: 2389.8755 - val_loss: 1951.8323 - val_mse: 1951.8323
Epoch 25/500
10/10 [==============================] - 0s 3ms/step - loss: 2292.4839 -
mse: 2292.4839 - val_loss: 1903.5187 - val_mse: 1903.5187
Epoch 26/500
10/10 [==============================] - 0s 4ms/step - loss: 2070.6101 -
mse: 2070.6101 - val_loss: 1854.6979 - val_mse: 1854.6979
Epoch 27/500
10/10 [==============================] - 0s 4ms/step - loss: 2080.5908 -
mse: 2080.5908 - val_loss: 1823.5801 - val_mse: 1823.5801
Epoch 28/500
10/10 [==============================] - 0s 4ms/step - loss: 2144.8110 -
mse: 2144.8110 - val_loss: 1783.2067 - val_mse: 1783.2067
Epoch 29/500
10/10 [==============================] - 0s 4ms/step - loss: 2347.6414 -
mse: 2347.6414 - val_loss: 1762.4703 - val_mse: 1762.4703
Epoch 30/500
10/10 [==============================] - 0s 4ms/step - loss: 1933.8788 -
mse: 1933.8788 - val_loss: 1732.2152 - val_mse: 1732.2152
Epoch 31/500
10/10 [==============================] - 0s 3ms/step - loss: 2120.7637 -
mse: 2120.7637 - val_loss: 1701.5151 - val_mse: 1701.5151
Epoch 32/500
10/10 [==============================] - 0s 4ms/step - loss: 2061.1006 -
mse: 2061.1006 - val_loss: 1666.3718 - val_mse: 1666.3718
Epoch 33/500
10/10 [==============================] - 0s 3ms/step - loss: 2004.1423 -
mse: 2004.1422 - val_loss: 1610.8424 - val_mse: 1610.8424
Epoch 34/500
10/10 [==============================] - 0s 4ms/step - loss: 2029.0154 -
mse: 2029.0154 - val_loss: 1566.4816 - val_mse: 1566.4816
Epoch 35/500
10/10 [==============================] - 0s 4ms/step - loss: 1673.5566 -
mse: 1673.5566 - val_loss: 1524.0061 - val_mse: 1524.0061
Epoch 36/500
10/10 [==============================] - 0s 4ms/step - loss: 1795.5920 -
mse: 1795.5920 - val_loss: 1496.8381 - val_mse: 1496.8381
Epoch 37/500
10/10 [==============================] - 0s 4ms/step - loss: 1723.1383 -
```

```
mse: 1723.1383 - val_loss: 1467.2522 - val_mse: 1467.2522
Epoch 38/500
10/10 [==============================] - 0s 4ms/step - loss: 1689.2614 -
mse: 1689.2614 - val_loss: 1436.6813 - val_mse: 1436.6813
Epoch 39/500
10/10 [==============================] - 0s 4ms/step - loss: 1865.7874 -
mse: 1865.7874 - val_loss: 1407.7626 - val_mse: 1407.7626
Epoch 40/500
10/10 [==============================] - 0s 4ms/step - loss: 2048.2585 -
mse: 2048.2585 - val_loss: 1390.5431 - val_mse: 1390.5431
Epoch 41/500
10/10 [==============================] - 0s 4ms/step - loss: 1706.8531 -
mse: 1706.8531 - val_loss: 1365.1997 - val_mse: 1365.1998
Epoch 42/500
10/10 [==============================] - 0s 3ms/step - loss: 1937.6060 -
mse: 1937.6060 - val_loss: 1335.3107 - val_mse: 1335.3107
Epoch 43/500
10/10 [==============================] - 0s 4ms/step - loss: 1601.1340 -
mse: 1601.1340 - val_loss: 1310.9661 - val_mse: 1310.9661
Epoch 44/500
10/10 [==============================] - 0s 4ms/step - loss: 1537.4478 -
mse: 1537.4478 - val_loss: 1285.3125 - val_mse: 1285.3125
Epoch 45/500
10/10 [==============================] - 0s 4ms/step - loss: 1827.6326 -
mse: 1827.6326 - val_loss: 1270.3694 - val_mse: 1270.3694
Epoch 46/500
10/10 [==============================] - 0s 5ms/step - loss: 1588.1027 -
mse: 1588.1025 - val_loss: 1273.9954 - val_mse: 1273.9954
Epoch 47/500
10/10 [==============================] - 0s 5ms/step - loss: 1682.6407 -
mse: 1682.6407 - val_loss: 1256.1118 - val_mse: 1256.1118
Epoch 48/500
10/10 [==============================] - 0s 4ms/step - loss: 1922.7532 -
mse: 1922.7532 - val_loss: 1245.0521 - val_mse: 1245.0522
Epoch 49/500
10/10 [==============================] - 0s 4ms/step - loss: 1584.2712 -
mse: 1584.2710 - val_loss: 1233.5823 - val_mse: 1233.5823
Epoch 50/500
10/10 [==============================] - 0s 3ms/step - loss: 1513.9280 -
mse: 1513.9280 - val_loss: 1210.9475 - val_mse: 1210.9475
Epoch 51/500
10/10 [==============================] - 0s 4ms/step - loss: 1816.0553 -
mse: 1816.0553 - val_loss: 1201.8634 - val_mse: 1201.8635
Epoch 52/500
10/10 [==============================] - 0s 4ms/step - loss: 1655.7990 -
mse: 1655.7990 - val_loss: 1178.0294 - val_mse: 1178.0294
Epoch 53/500
10/10 [==============================] - 0s 4ms/step - loss: 1641.3564 -
mse: 1641.3564 - val_loss: 1170.9902 - val_mse: 1170.9902
Epoch 54/500
10/10 [==============================] - 0s 4ms/step - loss: 1523.4384 -
mse: 1523.4384 - val_loss: 1164.1022 - val_mse: 1164.1022
Epoch 55/500
10/10 [==============================] - 0s 3ms/step - loss: 1480.5582 -
mse: 1480.5582 - val_loss: 1150.2328 - val_mse: 1150.2328
Epoch 56/500
10/10 [==============================] - 0s 4ms/step - loss: 1542.3590 -
mse: 1542.3590 - val_loss: 1133.5095 - val_mse: 1133.5095
Epoch 57/500
10/10 [==============================] - 0s 4ms/step - loss: 1388.3911 -
mse: 1388.3912 - val_loss: 1132.3831 - val_mse: 1132.3831
```

```
Epoch 58/500
10/10 [==============================] - 0s 4ms/step - loss: 1704.9537 -
mse: 1704.9537 - val_loss: 1126.0933 - val_mse: 1126.0933
Epoch 59/500
10/10 [==============================] - 0s 4ms/step - loss: 1501.8459 -
mse: 1501.8459 - val_loss: 1118.7814 - val_mse: 1118.7814
Epoch 60/500
10/10 [==============================] - 0s 4ms/step - loss: 1488.4066 -
mse: 1488.4066 - val_loss: 1116.7825 - val_mse: 1116.7825
Epoch 61/500
10/10 [==============================] - 0s 3ms/step - loss: 1612.2938 -
mse: 1612.2938 - val_loss: 1115.7092 - val_mse: 1115.7092
Epoch 62/500
10/10 [==============================] - 0s 4ms/step - loss: 1400.9644 -
mse: 1400.9644 - val_loss: 1113.2502 - val_mse: 1113.2502
Epoch 63/500
10/10 [==============================] - 0s 3ms/step - loss: 1481.7429 -
mse: 1481.7429 - val_loss: 1106.7555 - val_mse: 1106.7555
Epoch 64/500
10/10 [==============================] - 0s 4ms/step - loss: 1828.8519 -
mse: 1828.8519 - val_loss: 1107.2480 - val_mse: 1107.2480
Epoch 65/500
10/10 [==============================] - 0s 3ms/step - loss: 1409.8960 -
mse: 1409.8960 - val_loss: 1100.1747 - val_mse: 1100.1747
Epoch 66/500
10/10 [==============================] - 0s 3ms/step - loss: 1283.8092 -
mse: 1283.8093 - val_loss: 1101.1429 - val_mse: 1101.1429
Epoch 67/500
10/10 [==============================] - 0s 3ms/step - loss: 1605.5055 -
mse: 1605.5055 - val_loss: 1104.2782 - val_mse: 1104.2782
Epoch 68/500
10/10 [==============================] - 0s 4ms/step - loss: 1632.5492 -
mse: 1632.5492 - val_loss: 1111.7773 - val_mse: 1111.7773
Epoch 69/500
10/10 [==============================] - 0s 3ms/step - loss: 1674.4296 -
mse: 1674.4296 - val_loss: 1139.0167 - val_mse: 1139.0167
Epoch 70/500
10/10 [==============================] - 0s 3ms/step - loss: 1525.4738 -
mse: 1525.4738 - val_loss: 1109.5133 - val_mse: 1109.5133
Epoch 71/500
10/10 [==============================] - 0s 3ms/step - loss: 1716.6189 -
mse: 1716.6189 - val_loss: 1072.7677 - val_mse: 1072.7677
Epoch 72/500
10/10 [==============================] - 0s 3ms/step - loss: 1461.0848 -
mse: 1461.0848 - val_loss: 1064.0365 - val_mse: 1064.0365
Epoch 73/500
10/10 [==============================] - 0s 3ms/step - loss: 1624.7115 -
mse: 1624.7115 - val_loss: 1064.6389 - val_mse: 1064.6389
Epoch 74/500
10/10 [==============================] - 0s 3ms/step - loss: 1545.7759 -
mse: 1545.7759 - val_loss: 1064.7792 - val_mse: 1064.7792
Epoch 75/500
10/10 [==============================] - 0s 3ms/step - loss: 1617.8677 -
mse: 1617.8677 - val_loss: 1046.3124 - val_mse: 1046.3123
Epoch 76/500
10/10 [==============================] - 0s 4ms/step - loss: 1507.6062 -
mse: 1507.6062 - val_loss: 1057.3970 - val_mse: 1057.3970
Epoch 77/500
10/10 [==============================] - 0s 3ms/step - loss: 1636.3237 -
mse: 1636.3237 - val_loss: 1052.0903 - val_mse: 1052.0903
Epoch 78/500
```

```
10/10 [==============================] - 0s 3ms/step - loss: 1466.4041 -
mse: 1466.4041 - val_loss: 1042.3163 - val_mse: 1042.3163
Epoch 79/500
10/10 [==============================] - 0s 3ms/step - loss: 1599.5238 -
mse: 1599.5238 - val_loss: 1013.6000 - val_mse: 1013.6000
Epoch 80/500
10/10 [==============================] - 0s 3ms/step - loss: 1637.8413 -
mse: 1637.8413 - val_loss: 1021.3125 - val_mse: 1021.3125
Epoch 81/500
10/10 [==============================] - 0s 5ms/step - loss: 1325.9983 -
mse: 1325.9983 - val_loss: 1013.3209 - val_mse: 1013.3209
Epoch 82/500
10/10 [==============================] - 0s 5ms/step - loss: 1567.1573 -
mse: 1567.1573 - val_loss: 1008.1964 - val_mse: 1008.1964
Epoch 83/500
10/10 [==============================] - 0s 3ms/step - loss: 1443.4208 -
mse: 1443.4208 - val_loss: 1004.2158 - val_mse: 1004.2158
Epoch 84/500
10/10 [==============================] - 0s 3ms/step - loss: 1621.9407 -
mse: 1621.9407 - val_loss: 1013.2300 - val_mse: 1013.2300
Epoch 85/500
10/10 [==============================] - 0s 4ms/step - loss: 1332.0472 -
mse: 1332.0472 - val_loss: 1018.9466 - val_mse: 1018.9466
Epoch 86/500
10/10 [==============================] - 0s 3ms/step - loss: 1474.2871 -
mse: 1474.2871 - val_loss: 994.8481 - val_mse: 994.8481
Epoch 87/500
10/10 [==============================] - 0s 3ms/step - loss: 1393.5994 -
mse: 1393.5994 - val_loss: 984.1044 - val_mse: 984.1044
Epoch 88/500
10/10 [==============================] - 0s 4ms/step - loss: 1540.8494 -
mse: 1540.8494 - val_loss: 1000.8593 - val_mse: 1000.8593
Epoch 89/500
10/10 [==============================] - 0s 3ms/step - loss: 1397.4808 -
mse: 1397.4808 - val_loss: 996.7819 - val_mse: 996.7819
Epoch 90/500
10/10 [==============================] - 0s 4ms/step - loss: 1340.9755 -
mse: 1340.9753 - val_loss: 994.9642 - val_mse: 994.9642
Epoch 91/500
10/10 [==============================] - 0s 3ms/step - loss: 1388.3473 -
mse: 1388.3473 - val_loss: 1007.4903 - val_mse: 1007.4903
Epoch 92/500
10/10 [==============================] - 0s 4ms/step - loss: 1358.9313 -
mse: 1358.9313 - val_loss: 1000.5961 - val_mse: 1000.5961
Epoch 93/500
10/10 [==============================] - 0s 4ms/step - loss: 1337.6323 -
mse: 1337.6323 - val_loss: 983.3044 - val_mse: 983.3044
Epoch 94/500
10/10 [==============================] - 0s 3ms/step - loss: 1221.3855 -
mse: 1221.3855 - val_loss: 978.1163 - val_mse: 978.1163
Epoch 95/500
10/10 [==============================] - 0s 4ms/step - loss: 1660.2075 -
mse: 1660.2076 - val_loss: 977.7682 - val_mse: 977.7682
Epoch 96/500
10/10 [==============================] - 0s 3ms/step - loss: 1262.6306 -
mse: 1262.6306 - val_loss: 989.1768 - val_mse: 989.1768
Epoch 97/500
10/10 [==============================] - 0s 4ms/step - loss: 1441.6158 -
mse: 1441.6158 - val_loss: 989.9921 - val_mse: 989.9921
Epoch 98/500
10/10 [==============================] - 0s 3ms/step - loss: 1460.1200 -
```

```
mse: 1460.1200 - val_loss: 991.1652 - val_mse: 991.1652
Epoch 99/500
10/10 [==============================] - 0s 3ms/step - loss: 1291.0208 -
mse: 1291.0208 - val_loss: 987.1071 - val_mse: 987.1071
Epoch 100/500
10/10 [==============================] - 0s 3ms/step - loss: 1391.8069 -
mse: 1391.8069 - val_loss: 982.2734 - val_mse: 982.2734
Epoch 101/500
10/10 [==============================] - 0s 3ms/step - loss: 1423.8914 -
mse: 1423.8914 - val_loss: 972.9385 - val_mse: 972.9385
Epoch 102/500
10/10 [==============================] - 0s 3ms/step - loss: 1407.4174 -
mse: 1407.4174 - val_loss: 970.5777 - val_mse: 970.5777
Epoch 103/500
10/10 [==============================] - 0s 3ms/step - loss: 1328.5078 -
mse: 1328.5078 - val_loss: 968.5988 - val_mse: 968.5988
Epoch 104/500
10/10 [==============================] - 0s 4ms/step - loss: 1546.8564 -
mse: 1546.8564 - val_loss: 962.0967 - val_mse: 962.0967
Epoch 105/500
10/10 [==============================] - 0s 3ms/step - loss: 1397.0552 -
mse: 1397.0552 - val_loss: 952.6219 - val_mse: 952.6219
Epoch 106/500
10/10 [==============================] - 0s 4ms/step - loss: 1528.3878 -
mse: 1528.3878 - val_loss: 978.2203 - val_mse: 978.2202
Epoch 107/500
10/10 [==============================] - 0s 3ms/step - loss: 1548.9918 -
mse: 1548.9918 - val_loss: 971.5810 - val_mse: 971.5810
Epoch 108/500
10/10 [==============================] - 0s 3ms/step - loss: 1286.6968 -
mse: 1286.6968 - val_loss: 953.4104 - val_mse: 953.4104
Epoch 109/500
10/10 [==============================] - 0s 3ms/step - loss: 1370.1697 -
mse: 1370.1698 - val_loss: 946.2525 - val_mse: 946.2525
Epoch 110/500
10/10 [==============================] - 0s 4ms/step - loss: 1657.2716 -
mse: 1657.2716 - val_loss: 967.9604 - val_mse: 967.9604
Epoch 111/500
10/10 [==============================] - 0s 3ms/step - loss: 1484.2999 -
mse: 1484.2999 - val_loss: 963.3969 - val_mse: 963.3969
Epoch 112/500
10/10 [==============================] - 0s 3ms/step - loss: 1420.0858 -
mse: 1420.0858 - val_loss: 963.3353 - val_mse: 963.3353
Epoch 113/500
10/10 [==============================] - 0s 4ms/step - loss: 1653.5613 -
mse: 1653.5613 - val_loss: 957.5650 - val_mse: 957.5650
Epoch 114/500
10/10 [==============================] - 0s 4ms/step - loss: 1575.9922 -
mse: 1575.9922 - val_loss: 937.5743 - val_mse: 937.5743
Epoch 115/500
10/10 [==============================] - 0s 3ms/step - loss: 1383.3174 -
mse: 1383.3174 - val_loss: 923.6319 - val_mse: 923.6319
Epoch 116/500
10/10 [==============================] - 0s 3ms/step - loss: 1354.1208 -
mse: 1354.1208 - val_loss: 922.7648 - val_mse: 922.7648
Epoch 117/500
10/10 [==============================] - 0s 4ms/step - loss: 1392.3209 -
mse: 1392.3209 - val_loss: 929.3973 - val_mse: 929.3973
Epoch 118/500
10/10 [==============================] - 0s 4ms/step - loss: 1712.8008 -
mse: 1712.8008 - val_loss: 921.2775 - val_mse: 921.2774
```

```
Epoch 119/500
10/10 [==============================] - 0s 4ms/step - loss: 1504.9375 - 
mse: 1504.9375 - val_loss: 917.9805 - val_mse: 917.9805
Epoch 120/500
10/10 [==============================] - 0s 4ms/step - loss: 1209.1926 - 
mse: 1209.1926 - val_loss: 917.1066 - val_mse: 917.1066
Epoch 121/500
10/10 [==============================] - 0s 3ms/step - loss: 1532.2646 - 
mse: 1532.2645 - val_loss: 913.7321 - val_mse: 913.7321
Epoch 122/500
10/10 [==============================] - 0s 3ms/step - loss: 1374.5208 - 
mse: 1374.5208 - val_loss: 901.1774 - val_mse: 901.1773
Epoch 123/500
10/10 [==============================] - 0s 4ms/step - loss: 1402.3363 - 
mse: 1402.3363 - val_loss: 893.7114 - val_mse: 893.7114
Epoch 124/500
10/10 [==============================] - 0s 4ms/step - loss: 1449.3889 - 
mse: 1449.3889 - val_loss: 907.0013 - val_mse: 907.0013
Epoch 125/500
10/10 [==============================] - 0s 3ms/step - loss: 1443.4170 - 
mse: 1443.4170 - val_loss: 918.4592 - val_mse: 918.4592
Epoch 126/500
10/10 [==============================] - 0s 3ms/step - loss: 1502.7280 - 
mse: 1502.7279 - val_loss: 927.5403 - val_mse: 927.5403
Epoch 127/500
10/10 [==============================] - 0s 4ms/step - loss: 1225.1086 - 
mse: 1225.1086 - val_loss: 913.9559 - val_mse: 913.9559
Epoch 128/500
10/10 [==============================] - 0s 4ms/step - loss: 1066.6537 - 
mse: 1066.6537 - val_loss: 908.6097 - val_mse: 908.6097
Epoch 129/500
10/10 [==============================] - 0s 4ms/step - loss: 1431.9304 - 
mse: 1431.9304 - val_loss: 906.5583 - val_mse: 906.5583
Epoch 130/500
10/10 [==============================] - 0s 3ms/step - loss: 1373.9139 - 
mse: 1373.9139 - val_loss: 915.6204 - val_mse: 915.6204
Epoch 131/500
10/10 [==============================] - 0s 4ms/step - loss: 1414.6234 - 
mse: 1414.6234 - val_loss: 934.0437 - val_mse: 934.0437
Epoch 132/500
10/10 [==============================] - 0s 3ms/step - loss: 1304.5679 - 
mse: 1304.5679 - val_loss: 910.3835 - val_mse: 910.3835
Epoch 133/500
10/10 [==============================] - 0s 3ms/step - loss: 1398.8020 - 
mse: 1398.8020 - val_loss: 891.3629 - val_mse: 891.3629
Epoch 134/500
10/10 [==============================] - 0s 4ms/step - loss: 1283.0360 - 
mse: 1283.0360 - val_loss: 879.9183 - val_mse: 879.9183
Epoch 135/500
10/10 [==============================] - 0s 3ms/step - loss: 1419.1575 - 
mse: 1419.1575 - val_loss: 879.2272 - val_mse: 879.2272
Epoch 136/500
10/10 [==============================] - 0s 3ms/step - loss: 1449.8429 - 
mse: 1449.8429 - val_loss: 881.5363 - val_mse: 881.5363
Epoch 137/500
10/10 [==============================] - 0s 4ms/step - loss: 1299.3246 - 
mse: 1299.3246 - val_loss: 913.9308 - val_mse: 913.9308
Epoch 138/500
10/10 [==============================] - 0s 4ms/step - loss: 1421.0500 - 
mse: 1421.0500 - val_loss: 926.0457 - val_mse: 926.0457
Epoch 139/500
```

```
10/10 [==============================] - 0s 3ms/step - loss: 1459.9695 -
mse: 1459.9695 - val_loss: 924.1078 - val_mse: 924.1080
Epoch 140/500
10/10 [==============================] - 0s 4ms/step - loss: 1556.2072 -
mse: 1556.2072 - val_loss: 906.1376 - val_mse: 906.1376
Epoch 141/500
10/10 [==============================] - 0s 3ms/step - loss: 1340.1305 -
mse: 1340.1305 - val_loss: 887.2172 - val_mse: 887.2172
Epoch 142/500
10/10 [==============================] - 0s 3ms/step - loss: 1577.2697 -
mse: 1577.2697 - val_loss: 901.0797 - val_mse: 901.0795
Epoch 143/500
10/10 [==============================] - 0s 3ms/step - loss: 1308.5398 -
mse: 1308.5398 - val_loss: 884.1826 - val_mse: 884.1826
Epoch 144/500
10/10 [==============================] - 0s 3ms/step - loss: 1489.9871 -
mse: 1489.9871 - val_loss: 865.4738 - val_mse: 865.4738
Epoch 145/500
10/10 [==============================] - 0s 3ms/step - loss: 1452.3877 -
mse: 1452.3877 - val_loss: 862.9601 - val_mse: 862.9601
Epoch 146/500
10/10 [==============================] - 0s 4ms/step - loss: 1421.4149 -
mse: 1421.4149 - val_loss: 878.8065 - val_mse: 878.8065
Epoch 147/500
10/10 [==============================] - 0s 3ms/step - loss: 1489.0149 -
mse: 1489.0149 - val_loss: 865.7858 - val_mse: 865.7858
Epoch 148/500
10/10 [==============================] - 0s 3ms/step - loss: 1391.5923 -
mse: 1391.5923 - val_loss: 854.5676 - val_mse: 854.5676
Epoch 149/500
10/10 [==============================] - 0s 3ms/step - loss: 1227.0861 -
mse: 1227.0861 - val_loss: 855.2108 - val_mse: 855.2108
Epoch 150/500
10/10 [==============================] - 0s 4ms/step - loss: 1354.7725 -
mse: 1354.7725 - val_loss: 859.3278 - val_mse: 859.3278
Epoch 151/500
10/10 [==============================] - 0s 3ms/step - loss: 1268.0596 -
mse: 1268.0596 - val_loss: 856.2560 - val_mse: 856.2560
Epoch 152/500
10/10 [==============================] - 0s 4ms/step - loss: 1484.6818 -
mse: 1484.6818 - val_loss: 860.2535 - val_mse: 860.2535
Epoch 153/500
10/10 [==============================] - 0s 3ms/step - loss: 1288.9027 -
mse: 1288.9027 - val_loss: 858.0693 - val_mse: 858.0693
Epoch 154/500
10/10 [==============================] - 0s 3ms/step - loss: 1244.9432 -
mse: 1244.9432 - val_loss: 860.1612 - val_mse: 860.1612
Epoch 155/500
10/10 [==============================] - 0s 3ms/step - loss: 1387.1777 -
mse: 1387.1777 - val_loss: 858.9459 - val_mse: 858.9459
Epoch 156/500
10/10 [==============================] - 0s 3ms/step - loss: 1376.3082 -
mse: 1376.3082 - val_loss: 864.5803 - val_mse: 864.5804
Epoch 157/500
10/10 [==============================] - 0s 3ms/step - loss: 1428.9827 -
mse: 1428.9827 - val_loss: 881.0049 - val_mse: 881.0049
Epoch 158/500
10/10 [==============================] - 0s 3ms/step - loss: 1233.8821 -
mse: 1233.8821 - val_loss: 887.1057 - val_mse: 887.1057
Epoch 159/500
10/10 [==============================] - 0s 3ms/step - loss: 1198.9510 -
```

```
mse: 1198.9510 - val_loss: 885.4933 - val_mse: 885.4933
Epoch 160/500
10/10 [==============================] - 0s 4ms/step - loss: 1265.2518 -
mse: 1265.2517 - val_loss: 886.6837 - val_mse: 886.6837
Epoch 161/500
10/10 [==============================] - 0s 3ms/step - loss: 1329.1714 -
mse: 1329.1714 - val_loss: 885.4796 - val_mse: 885.4796
Epoch 162/500
10/10 [==============================] - 0s 3ms/step - loss: 1134.1901 -
mse: 1134.1901 - val_loss: 884.0490 - val_mse: 884.0489
Epoch 163/500
10/10 [==============================] - 0s 4ms/step - loss: 1441.9873 -
mse: 1441.9873 - val_loss: 879.8815 - val_mse: 879.8815
Epoch 164/500
10/10 [==============================] - 0s 4ms/step - loss: 1352.3486 -
mse: 1352.3486 - val_loss: 885.7129 - val_mse: 885.7129
Epoch 165/500
10/10 [==============================] - 0s 3ms/step - loss: 1352.5852 -
mse: 1352.5852 - val_loss: 884.2549 - val_mse: 884.2549
Epoch 166/500
10/10 [==============================] - 0s 4ms/step - loss: 1383.3458 -
mse: 1383.3458 - val_loss: 861.0209 - val_mse: 861.0209
Epoch 167/500
10/10 [==============================] - 0s 3ms/step - loss: 1233.0540 -
mse: 1233.0540 - val_loss: 845.3071 - val_mse: 845.3070
Epoch 168/500
10/10 [==============================] - 0s 3ms/step - loss: 1316.9757 -
mse: 1316.9757 - val_loss: 860.9841 - val_mse: 860.9841
Epoch 169/500
10/10 [==============================] - 0s 4ms/step - loss: 1242.9202 -
mse: 1242.9202 - val_loss: 875.6343 - val_mse: 875.6343
Epoch 170/500
10/10 [==============================] - 0s 4ms/step - loss: 1447.0190 -
mse: 1447.0190 - val_loss: 905.5622 - val_mse: 905.5622
Epoch 171/500
10/10 [==============================] - 0s 3ms/step - loss: 1388.5140 -
mse: 1388.5140 - val_loss: 909.6386 - val_mse: 909.6386
Epoch 172/500
10/10 [==============================] - 0s 3ms/step - loss: 1152.2657 -
mse: 1152.2655 - val_loss: 890.7497 - val_mse: 890.7497
Epoch 173/500
10/10 [==============================] - 0s 3ms/step - loss: 1249.5753 -
mse: 1249.5753 - val_loss: 876.5150 - val_mse: 876.5149
Epoch 174/500
10/10 [==============================] - 0s 3ms/step - loss: 1459.0714 -
mse: 1459.0714 - val_loss: 878.5928 - val_mse: 878.5928
Epoch 175/500
10/10 [==============================] - 0s 3ms/step - loss: 1339.4919 -
mse: 1339.4919 - val_loss: 896.9111 - val_mse: 896.9111
Epoch 176/500
10/10 [==============================] - 0s 4ms/step - loss: 1385.0999 -
mse: 1385.0999 - val_loss: 892.0883 - val_mse: 892.0883
Epoch 177/500
10/10 [==============================] - 0s 3ms/step - loss: 1265.0493 -
mse: 1265.0493 - val_loss: 871.9827 - val_mse: 871.9827
Epoch 178/500
10/10 [==============================] - 0s 3ms/step - loss: 1298.9851 -
mse: 1298.9851 - val_loss: 896.0568 - val_mse: 896.0568
Epoch 179/500
10/10 [==============================] - 0s 3ms/step - loss: 1326.9996 -
mse: 1326.9996 - val_loss: 883.8196 - val_mse: 883.8197
```

```
Epoch 180/500
10/10 [==============================] - 0s 3ms/step - loss: 1374.6691 -
mse: 1374.6691 - val_loss: 867.3156 - val_mse: 867.3157
Epoch 181/500
10/10 [==============================] - 0s 3ms/step - loss: 1558.8361 -
mse: 1558.8361 - val_loss: 855.7555 - val_mse: 855.7555
Epoch 182/500
10/10 [==============================] - 0s 4ms/step - loss: 1439.6289 -
mse: 1439.6289 - val_loss: 861.9581 - val_mse: 861.9581
Epoch 183/500
10/10 [==============================] - 0s 3ms/step - loss: 1302.5295 -
mse: 1302.5295 - val_loss: 869.3503 - val_mse: 869.3503
Epoch 184/500
10/10 [==============================] - 0s 3ms/step - loss: 1422.5402 -
mse: 1422.5402 - val_loss: 857.8502 - val_mse: 857.8503
Epoch 185/500
10/10 [==============================] - 0s 4ms/step - loss: 1315.0216 -
mse: 1315.0216 - val_loss: 862.6243 - val_mse: 862.6243
Epoch 186/500
10/10 [==============================] - 0s 3ms/step - loss: 1296.9012 -
mse: 1296.9012 - val_loss: 867.5463 - val_mse: 867.5463
Epoch 187/500
10/10 [==============================] - 0s 3ms/step - loss: 1504.5354 -
mse: 1504.5354 - val_loss: 886.3314 - val_mse: 886.3314
Epoch 188/500
10/10 [==============================] - 0s 3ms/step - loss: 1479.4441 -
mse: 1479.4441 - val_loss: 903.4374 - val_mse: 903.4375
Epoch 189/500
10/10 [==============================] - 0s 4ms/step - loss: 1344.3164 -
mse: 1344.3164 - val_loss: 894.2666 - val_mse: 894.2666
Epoch 190/500
10/10 [==============================] - 0s 3ms/step - loss: 1572.4877 -
mse: 1572.4877 - val_loss: 882.6621 - val_mse: 882.6621
Epoch 191/500
10/10 [==============================] - 0s 3ms/step - loss: 1507.2208 -
mse: 1507.2208 - val_loss: 867.5823 - val_mse: 867.5823
Epoch 192/500
10/10 [==============================] - 0s 3ms/step - loss: 1386.7654 -
mse: 1386.7654 - val_loss: 868.7402 - val_mse: 868.7402
Epoch 193/500
10/10 [==============================] - 0s 4ms/step - loss: 1275.4830 -
mse: 1275.4832 - val_loss: 860.8688 - val_mse: 860.8688
Epoch 194/500
10/10 [==============================] - 0s 3ms/step - loss: 1251.3755 -
mse: 1251.3755 - val_loss: 868.5746 - val_mse: 868.5746
Epoch 195/500
10/10 [==============================] - 0s 3ms/step - loss: 1254.7910 -
mse: 1254.7910 - val_loss: 871.3702 - val_mse: 871.3702
Epoch 196/500
10/10 [==============================] - 0s 3ms/step - loss: 1316.7433 -
mse: 1316.7432 - val_loss: 870.5876 - val_mse: 870.5876
Epoch 197/500
10/10 [==============================] - 0s 3ms/step - loss: 1188.2527 -
mse: 1188.2527 - val_loss: 862.9415 - val_mse: 862.9415
Epoch 198/500
10/10 [==============================] - 0s 4ms/step - loss: 1401.1288 -
mse: 1401.1288 - val_loss: 855.9550 - val_mse: 855.9550
Epoch 199/500
10/10 [==============================] - 0s 3ms/step - loss: 1342.7401 -
mse: 1342.7401 - val_loss: 867.8028 - val_mse: 867.8028
Epoch 200/500
```

```
10/10 [==============================] - 0s 3ms/step - loss: 1163.7144 -
mse: 1163.7144 - val_loss: 864.8398 - val_mse: 864.8398
Epoch 201/500
10/10 [==============================] - 0s 3ms/step - loss: 1339.8873 -
mse: 1339.8873 - val_loss: 860.2594 - val_mse: 860.2594
Epoch 202/500
10/10 [==============================] - 0s 3ms/step - loss: 1223.8658 -
mse: 1223.8658 - val_loss: 869.2097 - val_mse: 869.2098
Epoch 203/500
10/10 [==============================] - 0s 3ms/step - loss: 1209.3794 -
mse: 1209.3794 - val_loss: 863.1144 - val_mse: 863.1144
Epoch 204/500
10/10 [==============================] - 0s 3ms/step - loss: 1019.3250 -
mse: 1019.3250 - val_loss: 855.1335 - val_mse: 855.1335
Epoch 205/500
10/10 [==============================] - 0s 4ms/step - loss: 1351.8086 -
mse: 1351.8086 - val_loss: 851.9599 - val_mse: 851.9599
Epoch 206/500
10/10 [==============================] - 0s 4ms/step - loss: 1185.7971 -
mse: 1185.7971 - val_loss: 848.8350 - val_mse: 848.8350
Epoch 207/500
10/10 [==============================] - 0s 3ms/step - loss: 1452.3344 -
mse: 1452.3344 - val_loss: 855.2585 - val_mse: 855.2585
Epoch 208/500
10/10 [==============================] - 0s 4ms/step - loss: 1361.8721 -
mse: 1361.8721 - val_loss: 861.7148 - val_mse: 861.7148
Epoch 209/500
10/10 [==============================] - 0s 3ms/step - loss: 1462.9761 -
mse: 1462.9761 - val_loss: 865.3972 - val_mse: 865.3972
Epoch 210/500
10/10 [==============================] - 0s 4ms/step - loss: 1257.7411 -
mse: 1257.7411 - val_loss: 852.7991 - val_mse: 852.7991
Epoch 211/500
10/10 [==============================] - 0s 4ms/step - loss: 1280.5521 -
mse: 1280.5521 - val_loss: 840.0846 - val_mse: 840.0846
Epoch 212/500
10/10 [==============================] - 0s 3ms/step - loss: 1295.4407 -
mse: 1295.4407 - val_loss: 840.6210 - val_mse: 840.6210
Epoch 213/500
10/10 [==============================] - 0s 3ms/step - loss: 1190.5464 -
mse: 1190.5464 - val_loss: 830.2053 - val_mse: 830.2053
Epoch 214/500
10/10 [==============================] - 0s 3ms/step - loss: 1311.9160 -
mse: 1311.9161 - val_loss: 827.8940 - val_mse: 827.8941
Epoch 215/500
10/10 [==============================] - 0s 3ms/step - loss: 1255.1476 -
mse: 1255.1476 - val_loss: 825.0800 - val_mse: 825.0800
Epoch 216/500
10/10 [==============================] - 0s 3ms/step - loss: 1147.9735 -
mse: 1147.9735 - val_loss: 831.5011 - val_mse: 831.5011
Epoch 217/500
10/10 [==============================] - 0s 4ms/step - loss: 1329.0809 -
mse: 1329.0809 - val_loss: 870.5206 - val_mse: 870.5206
Epoch 218/500
10/10 [==============================] - 0s 3ms/step - loss: 1003.9725 -
mse: 1003.9725 - val_loss: 864.7944 - val_mse: 864.7945
Epoch 219/500
10/10 [==============================] - 0s 4ms/step - loss: 1371.3850 -
mse: 1371.3850 - val_loss: 847.4214 - val_mse: 847.4213
Epoch 220/500
10/10 [==============================] - 0s 3ms/step - loss: 1210.2960 -
```

```
mse: 1210.2960 - val_loss: 826.5558 - val_mse: 826.5558
Epoch 221/500
10/10 [==============================] - 0s 3ms/step - loss: 1377.2032 -
mse: 1377.2032 - val_loss: 822.4355 - val_mse: 822.4355
Epoch 222/500
10/10 [==============================] - 0s 3ms/step - loss: 1215.3501 -
mse: 1215.3501 - val_loss: 825.0261 - val_mse: 825.0261
Epoch 223/500
10/10 [==============================] - 0s 4ms/step - loss: 1511.2577 -
mse: 1511.2577 - val_loss: 843.2322 - val_mse: 843.2322
Epoch 224/500
10/10 [==============================] - 0s 3ms/step - loss: 1373.5980 -
mse: 1373.5980 - val_loss: 855.9205 - val_mse: 855.9205
Epoch 225/500
10/10 [==============================] - 0s 3ms/step - loss: 1399.3063 -
mse: 1399.3063 - val_loss: 851.8068 - val_mse: 851.8067
Epoch 226/500
10/10 [==============================] - 0s 3ms/step - loss: 1361.1022 -
mse: 1361.1022 - val_loss: 855.3065 - val_mse: 855.3065
Epoch 227/500
10/10 [==============================] - 0s 3ms/step - loss: 1319.0980 -
mse: 1319.0980 - val_loss: 850.7313 - val_mse: 850.7313
Epoch 228/500
10/10 [==============================] - 0s 4ms/step - loss: 1152.0831 -
mse: 1152.0834 - val_loss: 858.4952 - val_mse: 858.4952
Epoch 229/500
10/10 [==============================] - 0s 4ms/step - loss: 1164.2443 -
mse: 1164.2443 - val_loss: 873.7361 - val_mse: 873.7361
Epoch 230/500
10/10 [==============================] - 0s 4ms/step - loss: 1143.4404 -
mse: 1143.4404 - val_loss: 862.0007 - val_mse: 862.0007
Epoch 231/500
10/10 [==============================] - 0s 3ms/step - loss: 1159.9283 -
mse: 1159.9283 - val_loss: 841.3090 - val_mse: 841.3090
Epoch 232/500
10/10 [==============================] - 0s 3ms/step - loss: 1413.9930 -
mse: 1413.9930 - val_loss: 831.0272 - val_mse: 831.0272
Epoch 233/500
10/10 [==============================] - 0s 4ms/step - loss: 1302.8505 -
mse: 1302.8505 - val_loss: 841.2010 - val_mse: 841.2010
Epoch 234/500
10/10 [==============================] - 0s 4ms/step - loss: 1195.3823 -
mse: 1195.3823 - val_loss: 845.6125 - val_mse: 845.6125
Epoch 235/500
10/10 [==============================] - 0s 4ms/step - loss: 1220.6643 -
mse: 1220.6643 - val_loss: 853.8000 - val_mse: 853.8000
Epoch 236/500
10/10 [==============================] - 0s 3ms/step - loss: 1342.1431 -
mse: 1342.1429 - val_loss: 865.1339 - val_mse: 865.1339
Epoch 237/500
10/10 [==============================] - 0s 3ms/step - loss: 1396.1638 -
mse: 1396.1638 - val_loss: 858.6710 - val_mse: 858.6710
Epoch 238/500
10/10 [==============================] - 0s 3ms/step - loss: 1174.6650 -
mse: 1174.6650 - val_loss: 847.6964 - val_mse: 847.6964
Epoch 239/500
10/10 [==============================] - 0s 3ms/step - loss: 1180.6395 -
mse: 1180.6395 - val_loss: 842.6660 - val_mse: 842.6660
Epoch 240/500
10/10 [==============================] - 0s 3ms/step - loss: 1190.4381 -
mse: 1190.4381 - val_loss: 836.7794 - val_mse: 836.7794
```

```
Epoch 241/500
10/10 [==============================] - 0s 3ms/step - loss: 1281.0610 -
mse: 1281.0610 - val_loss: 849.2946 - val_mse: 849.2946
Epoch 242/500
10/10 [==============================] - 0s 3ms/step - loss: 1188.5765 -
mse: 1188.5765 - val_loss: 867.5338 - val_mse: 867.5338
Epoch 243/500
10/10 [==============================] - 0s 3ms/step - loss: 1293.9366 -
mse: 1293.9366 - val_loss: 864.8455 - val_mse: 864.8456
Epoch 244/500
10/10 [==============================] - 0s 3ms/step - loss: 1317.6503 -
mse: 1317.6503 - val_loss: 862.0087 - val_mse: 862.0087
Epoch 245/500
10/10 [==============================] - 0s 3ms/step - loss: 1244.4012 -
mse: 1244.4012 - val_loss: 861.5791 - val_mse: 861.5791
Epoch 246/500
10/10 [==============================] - 0s 3ms/step - loss: 1265.8544 -
mse: 1265.8544 - val_loss: 870.3431 - val_mse: 870.3431
Epoch 247/500
10/10 [==============================] - 0s 3ms/step - loss: 1175.0448 -
mse: 1175.0448 - val_loss: 854.2028 - val_mse: 854.2028
Epoch 248/500
10/10 [==============================] - 0s 4ms/step - loss: 1127.2874 -
mse: 1127.2875 - val_loss: 848.3626 - val_mse: 848.3626
Epoch 249/500
10/10 [==============================] - 0s 3ms/step - loss: 1370.5411 -
mse: 1370.5411 - val_loss: 852.5035 - val_mse: 852.5035
Epoch 250/500
10/10 [==============================] - 0s 3ms/step - loss: 928.0772 -
mse: 928.0772 - val_loss: 841.5650 - val_mse: 841.5649
Epoch 251/500
10/10 [==============================] - 0s 4ms/step - loss: 1287.8813 -
mse: 1287.8813 - val_loss: 834.6448 - val_mse: 834.6447
Epoch 252/500
10/10 [==============================] - 0s 3ms/step - loss: 1326.9921 -
mse: 1326.9921 - val_loss: 824.6928 - val_mse: 824.6928
Epoch 253/500
10/10 [==============================] - 0s 3ms/step - loss: 1326.9996 -
mse: 1326.9996 - val_loss: 832.4226 - val_mse: 832.4226
Epoch 254/500
10/10 [==============================] - 0s 4ms/step - loss: 1279.6475 -
mse: 1279.6475 - val_loss: 846.8766 - val_mse: 846.8766
Epoch 255/500
10/10 [==============================] - 0s 4ms/step - loss: 1202.6486 -
mse: 1202.6486 - val_loss: 842.2457 - val_mse: 842.2457
Epoch 256/500
10/10 [==============================] - 0s 3ms/step - loss: 1059.6678 -
mse: 1059.6678 - val_loss: 832.1921 - val_mse: 832.1920
Epoch 257/500
10/10 [==============================] - 0s 3ms/step - loss: 1224.0874 -
mse: 1224.0874 - val_loss: 829.1927 - val_mse: 829.1927
Epoch 258/500
10/10 [==============================] - 0s 3ms/step - loss: 1045.0554 -
mse: 1045.0554 - val_loss: 829.4417 - val_mse: 829.4417
Epoch 259/500
10/10 [==============================] - 0s 3ms/step - loss: 1190.7587 -
mse: 1190.7587 - val_loss: 835.0453 - val_mse: 835.0453
Epoch 260/500
10/10 [==============================] - 0s 3ms/step - loss: 1261.8019 -
mse: 1261.8019 - val_loss: 829.3152 - val_mse: 829.3152
Epoch 261/500
```

```
10/10 [==============================] - 0s 4ms/step - loss: 1335.2837 -
mse: 1335.2837 - val_loss: 813.1221 - val_mse: 813.1221
Epoch 262/500
10/10 [==============================] - 0s 3ms/step - loss: 1306.9502 -
mse: 1306.9502 - val_loss: 801.4858 - val_mse: 801.4858
Epoch 263/500
10/10 [==============================] - 0s 3ms/step - loss: 1124.2382 -
mse: 1124.2382 - val_loss: 809.5982 - val_mse: 809.5983
Epoch 264/500
10/10 [==============================] - 0s 3ms/step - loss: 1156.9906 -
mse: 1156.9906 - val_loss: 826.6782 - val_mse: 826.6782
Epoch 265/500
10/10 [==============================] - 0s 3ms/step - loss: 1183.1121 -
mse: 1183.1121 - val_loss: 821.8893 - val_mse: 821.8893
Epoch 266/500
10/10 [==============================] - 0s 3ms/step - loss: 1261.7040 -
mse: 1261.7040 - val_loss: 818.4193 - val_mse: 818.4193
Epoch 267/500
10/10 [==============================] - 0s 3ms/step - loss: 1258.7502 -
mse: 1258.7502 - val_loss: 815.9050 - val_mse: 815.9049
Epoch 268/500
10/10 [==============================] - 0s 3ms/step - loss: 1278.9153 -
mse: 1278.9152 - val_loss: 820.1682 - val_mse: 820.1682
Epoch 269/500
10/10 [==============================] - 0s 3ms/step - loss: 1110.6006 -
mse: 1110.6006 - val_loss: 825.6319 - val_mse: 825.6319
Epoch 270/500
10/10 [==============================] - 0s 4ms/step - loss: 1260.1766 -
mse: 1260.1766 - val_loss: 827.6699 - val_mse: 827.6699
Epoch 271/500
10/10 [==============================] - 0s 3ms/step - loss: 1298.0310 -
mse: 1298.0310 - val_loss: 836.5291 - val_mse: 836.5291
Epoch 272/500
10/10 [==============================] - 0s 3ms/step - loss: 1173.5892 -
mse: 1173.5892 - val_loss: 831.5912 - val_mse: 831.5912
Epoch 273/500
10/10 [==============================] - 0s 4ms/step - loss: 1340.1892 -
mse: 1340.1892 - val_loss: 833.4030 - val_mse: 833.4030
Epoch 274/500
10/10 [==============================] - 0s 3ms/step - loss: 1023.6900 -
mse: 1023.6900 - val_loss: 837.1066 - val_mse: 837.1066
Epoch 275/500
10/10 [==============================] - 0s 3ms/step - loss: 1246.8879 -
mse: 1246.8881 - val_loss: 840.0847 - val_mse: 840.0847
Epoch 276/500
10/10 [==============================] - 0s 3ms/step - loss: 1228.7980 -
mse: 1228.7980 - val_loss: 841.7810 - val_mse: 841.7810
Epoch 277/500
10/10 [==============================] - 0s 3ms/step - loss: 1260.9452 -
mse: 1260.9452 - val_loss: 852.9122 - val_mse: 852.9122
Epoch 278/500
10/10 [==============================] - 0s 4ms/step - loss: 1171.2617 -
mse: 1171.2617 - val_loss: 859.6183 - val_mse: 859.6183
Epoch 279/500
10/10 [==============================] - 0s 4ms/step - loss: 1153.5492 -
mse: 1153.5492 - val_loss: 843.9161 - val_mse: 843.9161
Epoch 280/500
10/10 [==============================] - 0s 3ms/step - loss: 1238.8279 -
mse: 1238.8279 - val_loss: 825.9778 - val_mse: 825.9778
Epoch 281/500
10/10 [==============================] - 0s 3ms/step - loss: 1151.6793 -
```

```
mse: 1151.6793 - val_loss: 820.2541 - val_mse: 820.2541
Epoch 282/500
10/10 [==============================] - 0s 3ms/step - loss: 1057.1432 -
mse: 1057.1432 - val_loss: 823.4752 - val_mse: 823.4752
Epoch 283/500
10/10 [==============================] - 0s 3ms/step - loss: 1216.0938 -
mse: 1216.0936 - val_loss: 838.4866 - val_mse: 838.4867
Epoch 284/500
10/10 [==============================] - 0s 3ms/step - loss: 1352.6813 -
mse: 1352.6813 - val_loss: 840.8395 - val_mse: 840.8395
Epoch 285/500
10/10 [==============================] - 0s 3ms/step - loss: 1100.9877 -
mse: 1100.9877 - val_loss: 829.7587 - val_mse: 829.7588
Epoch 286/500
10/10 [==============================] - 0s 4ms/step - loss: 1353.0990 -
mse: 1353.0990 - val_loss: 830.6356 - val_mse: 830.6356
Epoch 287/500
10/10 [==============================] - 0s 3ms/step - loss: 1137.6095 -
mse: 1137.6095 - val_loss: 830.8150 - val_mse: 830.8151
Epoch 288/500
10/10 [==============================] - 0s 3ms/step - loss: 1077.0940 -
mse: 1077.0940 - val_loss: 829.0135 - val_mse: 829.0136
Epoch 289/500
10/10 [==============================] - 0s 3ms/step - loss: 1146.0309 -
mse: 1146.0309 - val_loss: 813.4857 - val_mse: 813.4857
Epoch 290/500
10/10 [==============================] - 0s 3ms/step - loss: 1339.0360 -
mse: 1339.0360 - val_loss: 820.3376 - val_mse: 820.3376
Epoch 291/500
10/10 [==============================] - 0s 4ms/step - loss: 1153.4806 -
mse: 1153.4806 - val_loss: 832.6123 - val_mse: 832.6123
Epoch 292/500
10/10 [==============================] - 0s 4ms/step - loss: 1050.7019 -
mse: 1050.7019 - val_loss: 829.5646 - val_mse: 829.5646
Epoch 293/500
10/10 [==============================] - 0s 3ms/step - loss: 1056.8273 -
mse: 1056.8273 - val_loss: 820.6135 - val_mse: 820.6135
Epoch 294/500
10/10 [==============================] - 0s 3ms/step - loss: 1276.2838 -
mse: 1276.2838 - val_loss: 824.8275 - val_mse: 824.8275
Epoch 295/500
10/10 [==============================] - 0s 3ms/step - loss: 1157.8737 -
mse: 1157.8737 - val_loss: 828.8962 - val_mse: 828.8962
Epoch 296/500
10/10 [==============================] - 0s 4ms/step - loss: 1172.0887 -
mse: 1172.0887 - val_loss: 820.0857 - val_mse: 820.0857
Epoch 297/500
10/10 [==============================] - 0s 3ms/step - loss: 1303.7992 -
mse: 1303.7992 - val_loss: 822.0605 - val_mse: 822.0605
Epoch 298/500
10/10 [==============================] - 0s 4ms/step - loss: 1327.6718 -
mse: 1327.6718 - val_loss: 816.7233 - val_mse: 816.7233
Epoch 299/500
10/10 [==============================] - 0s 4ms/step - loss: 1366.3309 -
mse: 1366.3309 - val_loss: 798.6808 - val_mse: 798.6808
Epoch 300/500
10/10 [==============================] - 0s 4ms/step - loss: 1039.6008 -
mse: 1039.6008 - val_loss: 794.2091 - val_mse: 794.2091
Epoch 301/500
10/10 [==============================] - 0s 3ms/step - loss: 1039.4171 -
mse: 1039.4171 - val_loss: 794.9846 - val_mse: 794.9846
```

```
Epoch 302/500
10/10 [==============================] - 0s 3ms/step - loss: 1314.6012 - mse: 1314.6012 - val_loss: 797.7585 - val_mse: 797.7585
Epoch 303/500
10/10 [==============================] - 0s 3ms/step - loss: 998.8743 - mse: 998.8743 - val_loss: 814.4571 - val_mse: 814.4571
Epoch 304/500
10/10 [==============================] - 0s 3ms/step - loss: 1354.2029 - mse: 1354.2029 - val_loss: 820.5987 - val_mse: 820.5987
Epoch 305/500
10/10 [==============================] - 0s 3ms/step - loss: 1207.0583 - mse: 1207.0583 - val_loss: 812.2078 - val_mse: 812.2078
Epoch 306/500
10/10 [==============================] - 0s 3ms/step - loss: 1158.9941 - mse: 1158.9941 - val_loss: 816.6388 - val_mse: 816.6388
Epoch 307/500
10/10 [==============================] - 0s 3ms/step - loss: 1052.7490 - mse: 1052.7490 - val_loss: 817.0644 - val_mse: 817.0645
Epoch 308/500
10/10 [==============================] - 0s 4ms/step - loss: 1213.0792 - mse: 1213.0792 - val_loss: 805.3510 - val_mse: 805.3510
Epoch 309/500
10/10 [==============================] - 0s 3ms/step - loss: 1281.0632 - mse: 1281.0632 - val_loss: 808.9374 - val_mse: 808.9374
Epoch 310/500
10/10 [==============================] - 0s 3ms/step - loss: 1332.5762 - mse: 1332.5762 - val_loss: 814.0074 - val_mse: 814.0074
Epoch 311/500
10/10 [==============================] - 0s 4ms/step - loss: 1230.6361 - mse: 1230.6361 - val_loss: 814.1874 - val_mse: 814.1874
Epoch 312/500
10/10 [==============================] - 0s 3ms/step - loss: 1221.6390 - mse: 1221.6390 - val_loss: 816.8105 - val_mse: 816.8106
Epoch 313/500
10/10 [==============================] - 0s 4ms/step - loss: 1200.6006 - mse: 1200.6006 - val_loss: 820.0698 - val_mse: 820.0698
Epoch 314/500
10/10 [==============================] - 0s 3ms/step - loss: 1082.6265 - mse: 1082.6265 - val_loss: 802.6320 - val_mse: 802.6320
Epoch 315/500
10/10 [==============================] - 0s 3ms/step - loss: 1060.2323 - mse: 1060.2323 - val_loss: 786.9466 - val_mse: 786.9466
Epoch 316/500
10/10 [==============================] - 0s 3ms/step - loss: 1095.5291 - mse: 1095.5291 - val_loss: 787.2719 - val_mse: 787.2719
Epoch 317/500
10/10 [==============================] - 0s 4ms/step - loss: 1002.3761 - mse: 1002.3761 - val_loss: 796.8747 - val_mse: 796.8747
Epoch 318/500
10/10 [==============================] - 0s 3ms/step - loss: 1191.3297 - mse: 1191.3297 - val_loss: 806.7337 - val_mse: 806.7337
Epoch 319/500
10/10 [==============================] - 0s 4ms/step - loss: 1203.7147 - mse: 1203.7147 - val_loss: 813.3934 - val_mse: 813.3934
Epoch 320/500
10/10 [==============================] - 0s 4ms/step - loss: 1184.3374 - mse: 1184.3374 - val_loss: 805.4969 - val_mse: 805.4968
Epoch 321/500
10/10 [==============================] - 0s 4ms/step - loss: 1271.7708 - mse: 1271.7708 - val_loss: 797.8563 - val_mse: 797.8563
Epoch 322/500
```

```
10/10 [==============================] - 0s 4ms/step - loss: 1076.2130 -
mse: 1076.2130 - val_loss: 800.5266 - val_mse: 800.5266
Epoch 323/500
10/10 [==============================] - 0s 4ms/step - loss: 1357.1953 -
mse: 1357.1953 - val_loss: 824.9630 - val_mse: 824.9630
Epoch 324/500
10/10 [==============================] - 0s 4ms/step - loss: 1246.5920 -
mse: 1246.5920 - val_loss: 845.5712 - val_mse: 845.5712
Epoch 325/500
10/10 [==============================] - 0s 3ms/step - loss: 1411.5997 -
mse: 1411.5997 - val_loss: 830.8689 - val_mse: 830.8689
Epoch 326/500
10/10 [==============================] - 0s 4ms/step - loss: 1175.1978 -
mse: 1175.1978 - val_loss: 813.3328 - val_mse: 813.3327
Epoch 327/500
10/10 [==============================] - 0s 3ms/step - loss: 994.2802 -
mse: 994.2802 - val_loss: 820.3597 - val_mse: 820.3597
Epoch 328/500
10/10 [==============================] - 0s 3ms/step - loss: 1321.2168 -
mse: 1321.2168 - val_loss: 806.9978 - val_mse: 806.9978
Epoch 329/500
10/10 [==============================] - 0s 4ms/step - loss: 1209.3185 -
mse: 1209.3185 - val_loss: 801.4709 - val_mse: 801.4709
Epoch 330/500
10/10 [==============================] - 0s 4ms/step - loss: 1191.0791 -
mse: 1191.0791 - val_loss: 819.8051 - val_mse: 819.8051
Epoch 331/500
10/10 [==============================] - 0s 3ms/step - loss: 1062.7347 -
mse: 1062.7347 - val_loss: 826.8831 - val_mse: 826.8831
Epoch 332/500
10/10 [==============================] - 0s 4ms/step - loss: 1232.1384 -
mse: 1232.1384 - val_loss: 812.3884 - val_mse: 812.3884
Epoch 333/500
10/10 [==============================] - 0s 4ms/step - loss: 1055.9913 -
mse: 1055.9913 - val_loss: 812.1691 - val_mse: 812.1691
Epoch 334/500
10/10 [==============================] - 0s 3ms/step - loss: 1117.3418 -
mse: 1117.3418 - val_loss: 808.3013 - val_mse: 808.3013
Epoch 335/500
10/10 [==============================] - 0s 4ms/step - loss: 1184.5702 -
mse: 1184.5702 - val_loss: 803.0842 - val_mse: 803.0842
Epoch 336/500
10/10 [==============================] - 0s 4ms/step - loss: 1180.8801 -
mse: 1180.8801 - val_loss: 803.8497 - val_mse: 803.8497
Epoch 337/500
10/10 [==============================] - 0s 3ms/step - loss: 1203.1394 -
mse: 1203.1394 - val_loss: 810.4517 - val_mse: 810.4517
Epoch 338/500
10/10 [==============================] - 0s 4ms/step - loss: 1257.1722 -
mse: 1257.1722 - val_loss: 818.6924 - val_mse: 818.6924
Epoch 339/500
10/10 [==============================] - 0s 3ms/step - loss: 1315.2811 -
mse: 1315.2811 - val_loss: 808.0102 - val_mse: 808.0102
Epoch 340/500
10/10 [==============================] - 0s 4ms/step - loss: 1205.0730 -
mse: 1205.0730 - val_loss: 798.3851 - val_mse: 798.3851
Epoch 341/500
10/10 [==============================] - 0s 3ms/step - loss: 1201.2455 -
mse: 1201.2455 - val_loss: 798.6164 - val_mse: 798.6164
Epoch 342/500
10/10 [==============================] - 0s 4ms/step - loss: 1336.2716 -
```

```
mse: 1336.2716 - val_loss: 802.4638 - val_mse: 802.4638
Epoch 343/500
10/10 [==============================] - 0s 3ms/step - loss: 1161.3898 -
mse: 1161.3898 - val_loss: 811.5505 - val_mse: 811.5505
Epoch 344/500
10/10 [==============================] - 0s 4ms/step - loss: 996.8325 -
mse: 996.8325 - val_loss: 823.8195 - val_mse: 823.8196
Epoch 345/500
10/10 [==============================] - 0s 3ms/step - loss: 1138.2850 -
mse: 1138.2850 - val_loss: 807.6110 - val_mse: 807.6110
Epoch 346/500
10/10 [==============================] - 0s 4ms/step - loss: 1449.2290 -
mse: 1449.2290 - val_loss: 809.6674 - val_mse: 809.6674
Epoch 347/500
10/10 [==============================] - 0s 3ms/step - loss: 1207.6294 -
mse: 1207.6294 - val_loss: 814.3858 - val_mse: 814.3857
Epoch 348/500
10/10 [==============================] - 0s 3ms/step - loss: 1206.6893 -
mse: 1206.6893 - val_loss: 819.4108 - val_mse: 819.4108
Epoch 349/500
10/10 [==============================] - 0s 3ms/step - loss: 1189.2942 -
mse: 1189.2942 - val_loss: 804.2780 - val_mse: 804.2780
Epoch 350/500
10/10 [==============================] - 0s 3ms/step - loss: 1094.7075 -
mse: 1094.7075 - val_loss: 799.4223 - val_mse: 799.4223
Epoch 351/500
10/10 [==============================] - 0s 3ms/step - loss: 1231.5222 -
mse: 1231.5222 - val_loss: 811.1283 - val_mse: 811.1283
Epoch 352/500
10/10 [==============================] - 0s 4ms/step - loss: 1169.2552 -
mse: 1169.2552 - val_loss: 812.0147 - val_mse: 812.0147
Epoch 353/500
10/10 [==============================] - 0s 3ms/step - loss: 1235.1083 -
mse: 1235.1083 - val_loss: 801.2874 - val_mse: 801.2873
Epoch 354/500
10/10 [==============================] - 0s 3ms/step - loss: 1001.0513 -
mse: 1001.0513 - val_loss: 803.0843 - val_mse: 803.0843
Epoch 355/500
10/10 [==============================] - 0s 4ms/step - loss: 1199.3776 -
mse: 1199.3777 - val_loss: 801.7519 - val_mse: 801.7519
Epoch 356/500
10/10 [==============================] - 0s 3ms/step - loss: 1061.2872 -
mse: 1061.2872 - val_loss: 801.2396 - val_mse: 801.2396
Epoch 357/500
10/10 [==============================] - 0s 3ms/step - loss: 1192.9622 -
mse: 1192.9622 - val_loss: 808.9018 - val_mse: 808.9018
Epoch 358/500
10/10 [==============================] - 0s 3ms/step - loss: 1268.4240 -
mse: 1268.4240 - val_loss: 815.0911 - val_mse: 815.0911
Epoch 359/500
10/10 [==============================] - 0s 3ms/step - loss: 1184.0792 -
mse: 1184.0792 - val_loss: 803.1102 - val_mse: 803.1102
Epoch 360/500
10/10 [==============================] - 0s 4ms/step - loss: 1058.5814 -
mse: 1058.5814 - val_loss: 791.9011 - val_mse: 791.9011
Epoch 361/500
10/10 [==============================] - 0s 3ms/step - loss: 1063.5259 -
mse: 1063.5259 - val_loss: 789.9473 - val_mse: 789.9473
Epoch 362/500
10/10 [==============================] - 0s 3ms/step - loss: 1054.5519 -
mse: 1054.5519 - val_loss: 802.4183 - val_mse: 802.4184
```

```
Epoch 363/500
10/10 [==============================] - 0s 4ms/step - loss: 1219.8267 -
mse: 1219.8267 - val_loss: 820.2123 - val_mse: 820.2123
Epoch 364/500
10/10 [==============================] - 0s 3ms/step - loss: 1299.6559 -
mse: 1299.6559 - val_loss: 813.4269 - val_mse: 813.4269
Epoch 365/500
10/10 [==============================] - 0s 4ms/step - loss: 1155.9423 -
mse: 1155.9423 - val_loss: 821.0289 - val_mse: 821.0289
Epoch 366/500
10/10 [==============================] - 0s 3ms/step - loss: 1155.8665 -
mse: 1155.8665 - val_loss: 827.2766 - val_mse: 827.2766
Epoch 367/500
10/10 [==============================] - 0s 3ms/step - loss: 1064.1320 -
mse: 1064.1320 - val_loss: 818.2502 - val_mse: 818.2502
Epoch 368/500
10/10 [==============================] - 0s 4ms/step - loss: 1134.5988 -
mse: 1134.5988 - val_loss: 808.0295 - val_mse: 808.0295
Epoch 369/500
10/10 [==============================] - 0s 3ms/step - loss: 1139.8890 -
mse: 1139.8890 - val_loss: 806.7741 - val_mse: 806.7741
Epoch 370/500
10/10 [==============================] - 0s 7ms/step - loss: 1066.1418 -
mse: 1066.1418 - val_loss: 803.5637 - val_mse: 803.5637
Epoch 371/500
10/10 [==============================] - 0s 5ms/step - loss: 1217.4030 -
mse: 1217.4030 - val_loss: 806.9114 - val_mse: 806.9114
Epoch 372/500
10/10 [==============================] - 0s 5ms/step - loss: 1190.1543 -
mse: 1190.1543 - val_loss: 808.1813 - val_mse: 808.1813
Epoch 373/500
10/10 [==============================] - 0s 3ms/step - loss: 1133.5552 -
mse: 1133.5552 - val_loss: 805.8232 - val_mse: 805.8232
Epoch 374/500
10/10 [==============================] - 0s 7ms/step - loss: 1145.7202 -
mse: 1145.7202 - val_loss: 808.4034 - val_mse: 808.4033
Epoch 375/500
10/10 [==============================] - 0s 6ms/step - loss: 1114.9786 -
mse: 1114.9786 - val_loss: 807.0558 - val_mse: 807.0558
Epoch 376/500
10/10 [==============================] - 0s 8ms/step - loss: 1093.2220 -
mse: 1093.2219 - val_loss: 795.8323 - val_mse: 795.8323
Epoch 377/500
10/10 [==============================] - 0s 6ms/step - loss: 1213.8601 -
mse: 1213.8601 - val_loss: 805.2751 - val_mse: 805.2751
Epoch 378/500
10/10 [==============================] - 0s 4ms/step - loss: 1146.4760 -
mse: 1146.4760 - val_loss: 815.4200 - val_mse: 815.4200
Epoch 379/500
10/10 [==============================] - 0s 3ms/step - loss: 1110.9978 -
mse: 1110.9978 - val_loss: 818.1246 - val_mse: 818.1246
Epoch 380/500
10/10 [==============================] - 0s 3ms/step - loss: 1241.0819 -
mse: 1241.0819 - val_loss: 814.3586 - val_mse: 814.3586
Epoch 381/500
10/10 [==============================] - 0s 3ms/step - loss: 1185.2542 -
mse: 1185.2542 - val_loss: 815.9216 - val_mse: 815.9216
Epoch 382/500
10/10 [==============================] - 0s 3ms/step - loss: 1124.8364 -
mse: 1124.8364 - val_loss: 816.1571 - val_mse: 816.1572
Epoch 383/500
```

```
10/10 [==============================] - 0s 3ms/step - loss: 1088.7362 -
mse: 1088.7362 - val_loss: 809.3951 - val_mse: 809.3951
Epoch 384/500
10/10 [==============================] - 0s 3ms/step - loss: 983.6995 -
mse: 983.6995 - val_loss: 790.4709 - val_mse: 790.4709
Epoch 385/500
10/10 [==============================] - 0s 3ms/step - loss: 1220.7457 -
mse: 1220.7457 - val_loss: 783.2112 - val_mse: 783.2112
Epoch 386/500
10/10 [==============================] - 0s 3ms/step - loss: 938.3348 -
mse: 938.3348 - val_loss: 787.4726 - val_mse: 787.4726
Epoch 387/500
10/10 [==============================] - 0s 3ms/step - loss: 1023.1074 -
mse: 1023.1074 - val_loss: 783.1712 - val_mse: 783.1712
Epoch 388/500
10/10 [==============================] - 0s 3ms/step - loss: 1162.6793 -
mse: 1162.6793 - val_loss: 785.3778 - val_mse: 785.3778
Epoch 389/500
10/10 [==============================] - 0s 3ms/step - loss: 1242.9272 -
mse: 1242.9272 - val_loss: 789.0985 - val_mse: 789.0985
Epoch 390/500
10/10 [==============================] - 0s 3ms/step - loss: 1029.1829 -
mse: 1029.1829 - val_loss: 807.5937 - val_mse: 807.5937
Epoch 391/500
10/10 [==============================] - 0s 4ms/step - loss: 1085.9567 -
mse: 1085.9567 - val_loss: 813.8329 - val_mse: 813.8329
Epoch 392/500
10/10 [==============================] - 0s 5ms/step - loss: 1194.8918 -
mse: 1194.8918 - val_loss: 813.2701 - val_mse: 813.2701
Epoch 393/500
10/10 [==============================] - 0s 4ms/step - loss: 1118.8864 -
mse: 1118.8864 - val_loss: 816.8326 - val_mse: 816.8326
Epoch 394/500
10/10 [==============================] - 0s 4ms/step - loss: 1156.2191 -
mse: 1156.2191 - val_loss: 817.6494 - val_mse: 817.6494
Epoch 395/500
10/10 [==============================] - 0s 4ms/step - loss: 1146.4380 -
mse: 1146.4380 - val_loss: 817.4178 - val_mse: 817.4177
Epoch 396/500
10/10 [==============================] - 0s 3ms/step - loss: 1195.0771 -
mse: 1195.0771 - val_loss: 801.3273 - val_mse: 801.3273
Epoch 397/500
10/10 [==============================] - 0s 3ms/step - loss: 1043.1863 -
mse: 1043.1863 - val_loss: 797.4997 - val_mse: 797.4997
Epoch 398/500
10/10 [==============================] - 0s 3ms/step - loss: 1058.5714 -
mse: 1058.5714 - val_loss: 796.9303 - val_mse: 796.9304
Epoch 399/500
10/10 [==============================] - 0s 3ms/step - loss: 973.2616 -
mse: 973.2616 - val_loss: 791.4570 - val_mse: 791.4570
Epoch 400/500
10/10 [==============================] - 0s 3ms/step - loss: 1078.3273 -
mse: 1078.3273 - val_loss: 789.6120 - val_mse: 789.6119
Epoch 401/500
10/10 [==============================] - 0s 4ms/step - loss: 1155.0522 -
mse: 1155.0522 - val_loss: 791.2770 - val_mse: 791.2770
Epoch 402/500
10/10 [==============================] - 0s 4ms/step - loss: 1125.3690 -
mse: 1125.3690 - val_loss: 797.8479 - val_mse: 797.8479
Epoch 403/500
10/10 [==============================] - 0s 3ms/step - loss: 1145.2904 -
```

```
mse: 1145.2905 - val_loss: 798.4960 - val_mse: 798.4960
Epoch 404/500
10/10 [==============================] - 0s 3ms/step - loss: 976.9025 -
mse: 976.9025 - val_loss: 789.4398 - val_mse: 789.4398
Epoch 405/500
10/10 [==============================] - 0s 3ms/step - loss: 1176.8585 -
mse: 1176.8585 - val_loss: 781.3744 - val_mse: 781.3744
Epoch 406/500
10/10 [==============================] - 0s 3ms/step - loss: 1167.7886 -
mse: 1167.7886 - val_loss: 777.2293 - val_mse: 777.2293
Epoch 407/500
10/10 [==============================] - 0s 3ms/step - loss: 1214.5470 -
mse: 1214.5470 - val_loss: 772.9610 - val_mse: 772.9610
Epoch 408/500
10/10 [==============================] - 0s 3ms/step - loss: 1165.4738 -
mse: 1165.4738 - val_loss: 777.4131 - val_mse: 777.4131
Epoch 409/500
10/10 [==============================] - 0s 3ms/step - loss: 977.5445 -
mse: 977.5445 - val_loss: 788.3349 - val_mse: 788.3349
Epoch 410/500
10/10 [==============================] - 0s 3ms/step - loss: 1142.0365 -
mse: 1142.0365 - val_loss: 803.3785 - val_mse: 803.3785
Epoch 411/500
10/10 [==============================] - 0s 3ms/step - loss: 1145.3531 -
mse: 1145.3531 - val_loss: 795.9268 - val_mse: 795.9268
Epoch 412/500
10/10 [==============================] - 0s 3ms/step - loss: 1200.5378 -
mse: 1200.5378 - val_loss: 794.3070 - val_mse: 794.3069
Epoch 413/500
10/10 [==============================] - 0s 4ms/step - loss: 1131.7947 -
mse: 1131.7947 - val_loss: 800.1425 - val_mse: 800.1425
Epoch 414/500
10/10 [==============================] - 0s 3ms/step - loss: 1081.5817 -
mse: 1081.5817 - val_loss: 804.3916 - val_mse: 804.3916
Epoch 415/500
10/10 [==============================] - 0s 3ms/step - loss: 1232.0951 -
mse: 1232.0951 - val_loss: 808.3775 - val_mse: 808.3775
Epoch 416/500
10/10 [==============================] - 0s 5ms/step - loss: 964.2349 -
mse: 964.2349 - val_loss: 799.5624 - val_mse: 799.5625
Epoch 417/500
10/10 [==============================] - 0s 3ms/step - loss: 976.0324 -
mse: 976.0324 - val_loss: 794.0538 - val_mse: 794.0538
Epoch 418/500
10/10 [==============================] - 0s 3ms/step - loss: 1009.3166 -
mse: 1009.3166 - val_loss: 792.2978 - val_mse: 792.2978
Epoch 419/500
10/10 [==============================] - 0s 3ms/step - loss: 1130.5861 -
mse: 1130.5861 - val_loss: 789.8478 - val_mse: 789.8478
Epoch 420/500
10/10 [==============================] - 0s 3ms/step - loss: 1161.0481 -
mse: 1161.0481 - val_loss: 786.4928 - val_mse: 786.4928
Epoch 421/500
10/10 [==============================] - 0s 3ms/step - loss: 1133.1250 -
mse: 1133.1250 - val_loss: 795.1569 - val_mse: 795.1569
Epoch 422/500
10/10 [==============================] - 0s 3ms/step - loss: 1169.5471 -
mse: 1169.5471 - val_loss: 791.6471 - val_mse: 791.6471
Epoch 423/500
10/10 [==============================] - 0s 6ms/step - loss: 930.6017 -
mse: 930.6016 - val_loss: 787.2552 - val_mse: 787.2553
```

```
Epoch 424/500
10/10 [==============================] - 0s 8ms/step - loss: 1122.6971 -
mse: 1122.6973 - val_loss: 779.5197 - val_mse: 779.5197
Epoch 425/500
10/10 [==============================] - 0s 8ms/step - loss: 1114.4113 -
mse: 1114.4113 - val_loss: 780.4168 - val_mse: 780.4168
Epoch 426/500
10/10 [==============================] - 0s 8ms/step - loss: 984.7107 -
mse: 984.7107 - val_loss: 781.7316 - val_mse: 781.7315
Epoch 427/500
10/10 [==============================] - 0s 11ms/step - loss: 1240.2795
- mse: 1240.2795 - val_loss: 781.1065 - val_mse: 781.1066
Epoch 428/500
10/10 [==============================] - 0s 13ms/step - loss: 1161.2776
- mse: 1161.2776 - val_loss: 786.1332 - val_mse: 786.1332
Epoch 429/500
10/10 [==============================] - 0s 8ms/step - loss: 1232.0874 -
mse: 1232.0874 - val_loss: 792.0220 - val_mse: 792.0220
Epoch 430/500
10/10 [==============================] - 0s 3ms/step - loss: 1192.5592 -
mse: 1192.5592 - val_loss: 786.6135 - val_mse: 786.6135
Epoch 431/500
10/10 [==============================] - 0s 5ms/step - loss: 1084.6805 -
mse: 1084.6805 - val_loss: 790.5133 - val_mse: 790.5133
Epoch 432/500
10/10 [==============================] - 0s 6ms/step - loss: 1202.0599 -
mse: 1202.0599 - val_loss: 808.2574 - val_mse: 808.2573
Epoch 433/500
10/10 [==============================] - 0s 3ms/step - loss: 1053.9030 -
mse: 1053.9030 - val_loss: 804.4003 - val_mse: 804.4004
Epoch 434/500
10/10 [==============================] - 0s 4ms/step - loss: 1101.4019 -
mse: 1101.4019 - val_loss: 803.1624 - val_mse: 803.1624
Epoch 435/500
10/10 [==============================] - 0s 3ms/step - loss: 981.4302 -
mse: 981.4302 - val_loss: 796.0095 - val_mse: 796.0095
Epoch 436/500
10/10 [==============================] - 0s 3ms/step - loss: 1164.5314 -
mse: 1164.5314 - val_loss: 790.0574 - val_mse: 790.0574
Epoch 437/500
10/10 [==============================] - 0s 3ms/step - loss: 1033.2937 -
mse: 1033.2937 - val_loss: 788.9647 - val_mse: 788.9648
Epoch 438/500
10/10 [==============================] - 0s 3ms/step - loss: 1328.4108 -
mse: 1328.4108 - val_loss: 800.1033 - val_mse: 800.1033
Epoch 439/500
10/10 [==============================] - 0s 3ms/step - loss: 1063.8362 -
mse: 1063.8362 - val_loss: 814.4783 - val_mse: 814.4783
Epoch 440/500
10/10 [==============================] - 0s 4ms/step - loss: 1138.3688 -
mse: 1138.3688 - val_loss: 810.5266 - val_mse: 810.5267
Epoch 441/500
10/10 [==============================] - 0s 3ms/step - loss: 1116.8839 -
mse: 1116.8839 - val_loss: 809.0814 - val_mse: 809.0814
Epoch 442/500
10/10 [==============================] - 0s 3ms/step - loss: 1121.9489 -
mse: 1121.9489 - val_loss: 797.3440 - val_mse: 797.3440
Epoch 443/500
10/10 [==============================] - 0s 3ms/step - loss: 1222.1353 -
mse: 1222.1353 - val_loss: 809.8318 - val_mse: 809.8318
Epoch 444/500
```

```
10/10 [==============================] - 0s 3ms/step - loss: 1210.7750 -
mse: 1210.7750 - val_loss: 809.8658 - val_mse: 809.8656
Epoch 445/500
10/10 [==============================] - 0s 3ms/step - loss: 1168.1641 -
mse: 1168.1641 - val_loss: 801.2050 - val_mse: 801.2050
Epoch 446/500
10/10 [==============================] - 0s 3ms/step - loss: 957.4722 -
mse: 957.4722 - val_loss: 796.3691 - val_mse: 796.3691
Epoch 447/500
10/10 [==============================] - 0s 3ms/step - loss: 1253.4639 -
mse: 1253.4639 - val_loss: 785.2164 - val_mse: 785.2164
Epoch 448/500
10/10 [==============================] - 0s 3ms/step - loss: 1134.3217 -
mse: 1134.3217 - val_loss: 786.2103 - val_mse: 786.2103
Epoch 449/500
10/10 [==============================] - 0s 3ms/step - loss: 1166.6348 -
mse: 1166.6348 - val_loss: 788.1888 - val_mse: 788.1888
Epoch 450/500
10/10 [==============================] - 0s 3ms/step - loss: 1197.9979 -
mse: 1197.9979 - val_loss: 794.4749 - val_mse: 794.4749
Epoch 451/500
10/10 [==============================] - 0s 3ms/step - loss: 972.3656 -
mse: 972.3656 - val_loss: 798.4323 - val_mse: 798.4323
Epoch 452/500
10/10 [==============================] - 0s 3ms/step - loss: 1097.3334 -
mse: 1097.3334 - val_loss: 793.3473 - val_mse: 793.3473
Epoch 453/500
10/10 [==============================] - 0s 3ms/step - loss: 1080.7708 -
mse: 1080.7708 - val_loss: 792.4955 - val_mse: 792.4955
Epoch 454/500
10/10 [==============================] - 0s 3ms/step - loss: 1055.2426 -
mse: 1055.2426 - val_loss: 794.6151 - val_mse: 794.6151
Epoch 455/500
10/10 [==============================] - 0s 3ms/step - loss: 1165.9719 -
mse: 1165.9719 - val_loss: 806.7936 - val_mse: 806.7938
Epoch 456/500
10/10 [==============================] - 0s 3ms/step - loss: 1161.9626 -
mse: 1161.9626 - val_loss: 811.5147 - val_mse: 811.5147
Epoch 457/500
10/10 [==============================] - 0s 4ms/step - loss: 1324.6805 -
mse: 1324.6805 - val_loss: 808.2443 - val_mse: 808.2443
Epoch 458/500
10/10 [==============================] - 0s 5ms/step - loss: 1137.2461 -
mse: 1137.2461 - val_loss: 786.8232 - val_mse: 786.8233
Epoch 459/500
10/10 [==============================] - 0s 3ms/step - loss: 1020.2510 -
mse: 1020.2509 - val_loss: 781.9742 - val_mse: 781.9742
Epoch 460/500
10/10 [==============================] - 0s 3ms/step - loss: 1039.8047 -
mse: 1039.8047 - val_loss: 785.2715 - val_mse: 785.2715
Epoch 461/500
10/10 [==============================] - 0s 3ms/step - loss: 1260.5406 -
mse: 1260.5405 - val_loss: 792.9179 - val_mse: 792.9179
Epoch 462/500
10/10 [==============================] - 0s 3ms/step - loss: 1222.4116 -
mse: 1222.4116 - val_loss: 812.8984 - val_mse: 812.8984
Epoch 463/500
10/10 [==============================] - 0s 3ms/step - loss: 1156.8169 -
mse: 1156.8169 - val_loss: 818.9448 - val_mse: 818.9448
Epoch 464/500
10/10 [==============================] - 0s 3ms/step - loss: 1170.2874 -
```

```
mse: 1170.2874 - val_loss: 815.9707 - val_mse: 815.9707
Epoch 465/500
10/10 [==============================] - 0s 4ms/step - loss: 1075.3651 -
mse: 1075.3651 - val_loss: 804.7527 - val_mse: 804.7526
Epoch 466/500
10/10 [==============================] - 0s 4ms/step - loss: 1047.0624 -
mse: 1047.0624 - val_loss: 794.8915 - val_mse: 794.8917
Epoch 467/500
10/10 [==============================] - 0s 3ms/step - loss: 1044.4875 -
mse: 1044.4875 - val_loss: 791.4354 - val_mse: 791.4353
Epoch 468/500
10/10 [==============================] - 0s 3ms/step - loss: 1201.2048 -
mse: 1201.2048 - val_loss: 790.7375 - val_mse: 790.7375
Epoch 469/500
10/10 [==============================] - 0s 3ms/step - loss: 1096.8907 -
mse: 1096.8907 - val_loss: 790.2315 - val_mse: 790.2316
Epoch 470/500
10/10 [==============================] - 0s 3ms/step - loss: 1005.1512 -
mse: 1005.1512 - val_loss: 792.1317 - val_mse: 792.1317
Epoch 471/500
10/10 [==============================] - 0s 3ms/step - loss: 1107.7635 -
mse: 1107.7635 - val_loss: 793.7138 - val_mse: 793.7138
Epoch 472/500
10/10 [==============================] - 0s 3ms/step - loss: 1246.3195 -
mse: 1246.3195 - val_loss: 818.2688 - val_mse: 818.2688
Epoch 473/500
10/10 [==============================] - 0s 3ms/step - loss: 1099.0110 -
mse: 1099.0110 - val_loss: 820.0930 - val_mse: 820.0930
Epoch 474/500
10/10 [==============================] - 0s 3ms/step - loss: 1081.6028 -
mse: 1081.6028 - val_loss: 813.3359 - val_mse: 813.3360
Epoch 475/500
10/10 [==============================] - 0s 3ms/step - loss: 1144.5924 -
mse: 1144.5924 - val_loss: 809.0876 - val_mse: 809.0876
Epoch 476/500
10/10 [==============================] - 0s 8ms/step - loss: 1088.8469 -
mse: 1088.8469 - val_loss: 805.6154 - val_mse: 805.6154
Epoch 477/500
10/10 [==============================] - 0s 4ms/step - loss: 1056.8949 -
mse: 1056.8949 - val_loss: 796.8518 - val_mse: 796.8518
Epoch 478/500
10/10 [==============================] - 0s 3ms/step - loss: 1093.0316 -
mse: 1093.0316 - val_loss: 791.3844 - val_mse: 791.3844
Epoch 479/500
10/10 [==============================] - 0s 3ms/step - loss: 1075.4176 -
mse: 1075.4176 - val_loss: 792.5369 - val_mse: 792.5370
Epoch 480/500
10/10 [==============================] - 0s 3ms/step - loss: 1040.8429 -
mse: 1040.8429 - val_loss: 799.0674 - val_mse: 799.0674
Epoch 481/500
10/10 [==============================] - 0s 3ms/step - loss: 1067.9944 -
mse: 1067.9944 - val_loss: 808.0246 - val_mse: 808.0246
Epoch 482/500
10/10 [==============================] - 0s 3ms/step - loss: 1075.4374 -
mse: 1075.4374 - val_loss: 804.6923 - val_mse: 804.6922
Epoch 483/500
10/10 [==============================] - 0s 3ms/step - loss: 1068.7610 -
mse: 1068.7610 - val_loss: 798.7229 - val_mse: 798.7229
Epoch 484/500
10/10 [==============================] - 0s 3ms/step - loss: 1096.5730 -
mse: 1096.5730 - val_loss: 800.5343 - val_mse: 800.5343
```

```
Epoch 485/500
10/10 [==============================] - 0s 3ms/step - loss: 928.6865 -
mse: 928.6865 - val_loss: 806.6980 - val_mse: 806.6980
Epoch 486/500
10/10 [==============================] - 0s 3ms/step - loss: 1073.0360 -
mse: 1073.0360 - val_loss: 812.5322 - val_mse: 812.5322
Epoch 487/500
10/10 [==============================] - 0s 3ms/step - loss: 956.9052 -
mse: 956.9052 - val_loss: 815.7873 - val_mse: 815.7873
Epoch 488/500
10/10 [==============================] - 0s 3ms/step - loss: 953.1597 -
mse: 953.1597 - val_loss: 808.8151 - val_mse: 808.8151
Epoch 489/500
10/10 [==============================] - 0s 3ms/step - loss: 1139.2506 -
mse: 1139.2506 - val_loss: 809.1796 - val_mse: 809.1796
Epoch 490/500
10/10 [==============================] - 0s 3ms/step - loss: 1151.7269 -
mse: 1151.7269 - val_loss: 811.4813 - val_mse: 811.4813
Epoch 491/500
10/10 [==============================] - 0s 3ms/step - loss: 1162.4410 -
mse: 1162.4409 - val_loss: 810.0572 - val_mse: 810.0572
Epoch 492/500
10/10 [==============================] - 0s 4ms/step - loss: 1176.6395 -
mse: 1176.6395 - val_loss: 815.9011 - val_mse: 815.9010
Epoch 493/500
10/10 [==============================] - 0s 3ms/step - loss: 953.2958 -
mse: 953.2958 - val_loss: 808.7178 - val_mse: 808.7178
Epoch 494/500
10/10 [==============================] - 0s 3ms/step - loss: 977.4135 -
mse: 977.4135 - val_loss: 800.9415 - val_mse: 800.9414
Epoch 495/500
10/10 [==============================] - 0s 3ms/step - loss: 1046.8663 -
mse: 1046.8663 - val_loss: 803.9940 - val_mse: 803.9940
Epoch 496/500
10/10 [==============================] - 0s 3ms/step - loss: 1065.1947 -
mse: 1065.1947 - val_loss: 803.8033 - val_mse: 803.8033
Epoch 497/500
10/10 [==============================] - 0s 4ms/step - loss: 1172.2582 -
mse: 1172.2582 - val_loss: 806.2042 - val_mse: 806.2042
Epoch 498/500
10/10 [==============================] - 0s 3ms/step - loss: 991.2426 -
mse: 991.2426 - val_loss: 809.2444 - val_mse: 809.2444
Epoch 499/500
10/10 [==============================] - 0s 3ms/step - loss: 853.4010 -
mse: 853.4010 - val_loss: 807.4279 - val_mse: 807.4279
Epoch 500/500
10/10 [==============================] - 0s 4ms/step - loss: 951.5442 -
mse: 951.5442 - val_loss: 808.1580 - val_mse: 808.1579
Neural Network: MSE: 748.5630257841979, EV: 0.5421572205772132
```
