# Supplementary material for: Generation and validation of a formula to calculate hemoglobin loss on a cohort of healthy adults subjected to controlled blood loss
Source: J Transl Med. 2021 Mar 20;19:116. doi: 10.1186/s12967-021-02783-9 (PMC7981850; doi:10.1186/s12967-021-02783-9)
Supplement: Supplementary file 3 — Additional file 3. Linear Regression [file 12967_2021_2783_MOESM3_ESM.pdf]

Blood loss prediction - Linear regression

Import

In [1]:
```
import pandas as pd
import numpy as np
from sklearn.model_selection import train_test_split, KFold
from sklearn.preprocessing import StandardScaler
from sklearn.metrics import mean_squared_error, mean_absolute_error, r2_
score, explained_variance_score
import matplotlib.pyplot as plt
import seaborn as sn
import statsmodels.api as sm
import statsmodels.stats.api as sms
from statsmodels.compat import lzip
from statsmodels.stats.outliers_influence import variance_inflation_fact
or
from statsmodels.stats.stattools import durbin_watson
from statsmodels.formula.api import rlm, ols
from scipy import stats

import warnings
warnings.filterwarnings("ignore")
```

Preprocessing

Data import

In [2]:
```
raw_data = pd.read_excel('Blood_loss_raw_data.xlsx')
raw_data['HB_diff'] = raw_data['HB_pre'] - raw_data['HB_post']
raw_data['BMI'] = raw_data['weight']/((raw_data['height']/100)**2)
raw_data.drop(['weight', 'height', 'BMI', 'age', 'HB_pre', 'HB_post', '
HB_diff', 'HCT_pre', 'HCT_post', 'BBS4PLT', 'BBS4LEUCO2', 'Nadler', 'VU_
Eisen','VU_Ferritin', 'loss_eryl', 'loss_ml'], inplace=True, axis=1)
raw_data.dropna(inplace=True)
raw_data = raw_data.sample(frac=1).reset_index()
raw_data
```

Out[2]:

	index	sex	BBS1PLT	BBS1LEUCO	BV_HB	loss_g
0	301	1	246	6.62	116.790975	129.727883
1	275	1	210	9.95	133.369391	156.281122
2	251	1	227	5.32	141.550780	127.570047
3	247	1	237	5.15	121.227672	123.303421
4	175	1	314	6.66	159.419890	105.929284
...	...	...	...	...	...	...

397	374	1	199	5.10	176.670213	165.787588
398	63	1	257	7.15	174.006751	91.125000
399	320	1	242	8.11	203.046809	150.990991
400	123	1	214	4.28	132.056957	123.992195
401	386	1	293	3.81	169.899696	161.286377

402 rows × 6 columns

Data transformations & visualizations

In [3]:
```
# BV_HB
raw_data['BV_HB'].plot.hist(grid=True, bins=20, rwidth=0.9, color='#607
c8e')
plt.title('Distribution of BV_HB')

# 95% value range
temp = list(raw_data['BV_HB'])
temp.sort()
print('Median: {} \n Mean: {}'.format(np.median(temp), np.mean(temp)))
print('95% of values between {} and {}'.format(temp[int(len(temp)*0.025
)], temp[int(len(temp)*0.975)]))
```

```
Median: 128.27970536010002
 Mean: 130.92171760715175
95% of values between 44.913212299999984 and 242.1822760048001
```

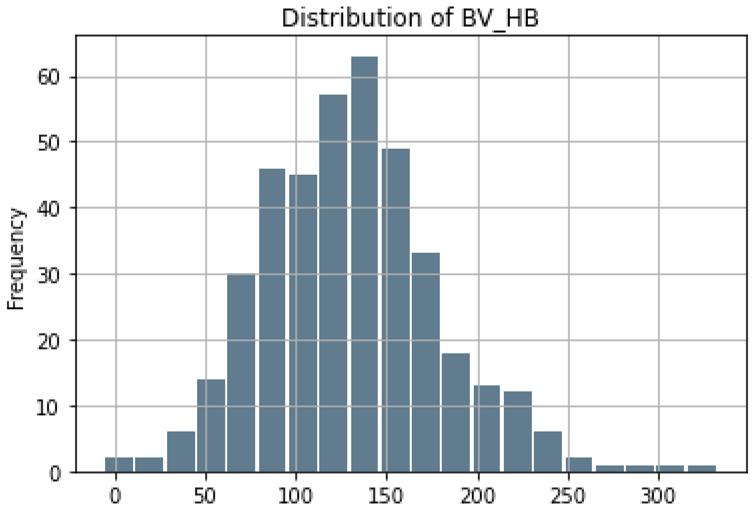

In [4]:
```
# blood loss (g)
raw_data['loss_g'].plot.hist(grid=True, bins=20, rwidth=0.9, color='#60
7c8e')
plt.title('Distribution of loss_g')
```

Out[4]: `Text(0.5, 1.0, 'Distribution of loss_g')`

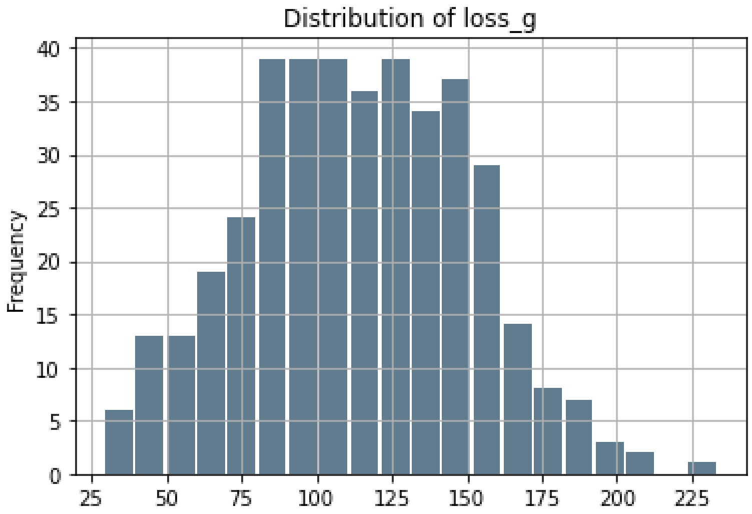

Data transformations

In [5]:
```
y = raw_data['loss_g']
X = raw_data.drop('loss_g', axis=1)

X_all = X[['sex', 'BV_HB']]
X_med = pd.DataFrame(X['BV_HB'])
```

Correlation matrix

In [6]:
```
X_temp = X[['BV_HB']]
X_temp['loss_g'] = y
cm = X_temp.corr()
sn.heatmap(cm, annot=True, cmap='Reds', fmt='.2g')
plt.show()
```

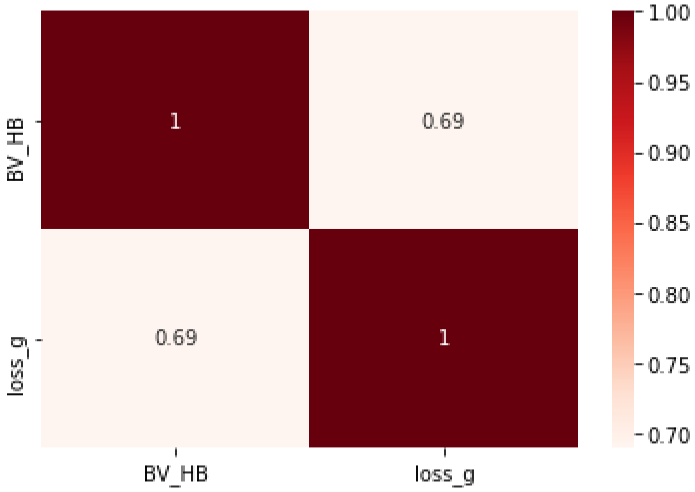

Medical model

In [7]:
```
#scaler = StandardScaler()
#X_med = scaler.fit_transform(X_med)
X_med2 = sm.add_constant(X_med)
est = sm.OLS(y, X_med2).fit()
print('Medical Model')
```

```
print(est.summary())
print('Condition number: {}'.format(est.condition_number))
```

```
Medical Model
                            OLS Regression Results

==========================================================================
======
Dep. Variable:                 loss_g   R-squared:                       0
.476
Model:                            OLS   Adj. R-squared:                  0
.475
Method:                 Least Squares   F-statistic:                     3
63.8
Date:                Thu, 01 Oct 2020   Prob (F-statistic):            3.7
8e-58
Time:                        11:19:15   Log-Likelihood:                -18
92.3
No. Observations:                 402   AIC:                             3
789.
Df Residuals:                     400   BIC:                            37
97.
Df Model:                           1

Covariance Type:            nonrobust

==========================================================================
======
                 coef    std err          t      P>|t|      [0.025      0.
975]
--------------------------------------------------------------------------
----------
const         45.7931      3.809     12.024      0.000      38.306      53
.280
BV_HB          0.5194      0.027     19.073      0.000       0.466       0
.573
==========================================================================
======
Omnibus:                        2.441   Durbin-Watson:                   2
.125
Prob(Omnibus):                  0.295   Jarque-Bera (JB):                2
.461
Skew:                           0.057   Prob(JB):                       0.
292
Kurtosis:                       3.366   Cond. No.
398.
==========================================================================
======

Warnings:
[1] Standard Errors assume that the covariance matrix of the errors is c
orrectly specified.
Condition number: 397.62149457636224
```

Linear regression model

In [8]:

```
# condition number issues disappears once variables are scaled
#scaler = StandardScaler()
#X_alls = scaler.fit_transform(X_all)
```

```
#X_all2 = sm.add_constant(X_alls)
X_all2 = sm.add_constant(X_all)
est = sm.OLS(y, X_all2).fit()
print(est.summary())

# Mean absolute error
print('Mean absolute error: {}'.format(sum(abs(est.resid))/len(est.res
id)))
```

```
                            OLS Regression Results

==============================================================================
======
Dep. Variable:                 loss_g   R-squared:                       0
.532
Model:                            OLS   Adj. R-squared:                  0
.530
Method:                 Least Squares   F-statistic:                     2
26.6
Date:                Thu, 01 Oct 2020   Prob (F-statistic):            1.7
4e-66
Time:                        11:19:15   Log-Likelihood:                -18
69.7
No. Observations:                 402   AIC:                             3
745.
Df Residuals:                     399   BIC:                            37
57.
Df Model:                           2

Covariance Type:            nonrobust

==============================================================================
======
                coef    std err          t      P>|t|      [0.025      0.
975]
------------------------------------------------------------------------------
----------
const        42.2123      3.643     11.588      0.000      35.051      49
.374
sex          19.5549      2.842      6.881      0.000      13.968      25
.142
BV_HB         0.4505      0.028     16.291      0.000       0.396       0
.505
==============================================================================
======
Omnibus:                       13.294   Durbin-Watson:                   2
.197
Prob(Omnibus):                  0.001   Jarque-Bera (JB):               17
.111
Skew:                          -0.307   Prob(JB):                     0.00
0193
Kurtosis:                       3.803   Cond. No.
408.
==============================================================================
======

Warnings:
[1] Standard Errors assume that the covariance matrix of the errors is c
orrectly specified.
Mean absolute error: 19.633425690017877
```

In [9]:
```
plt.scatter(est.predict(X_all2), est.resid)
plt.title("Residuals vs. predicted HB loss")
plt.xlabel("predicted HB loss (g)")
plt.ylabel("Residuals")
plt.show()
```

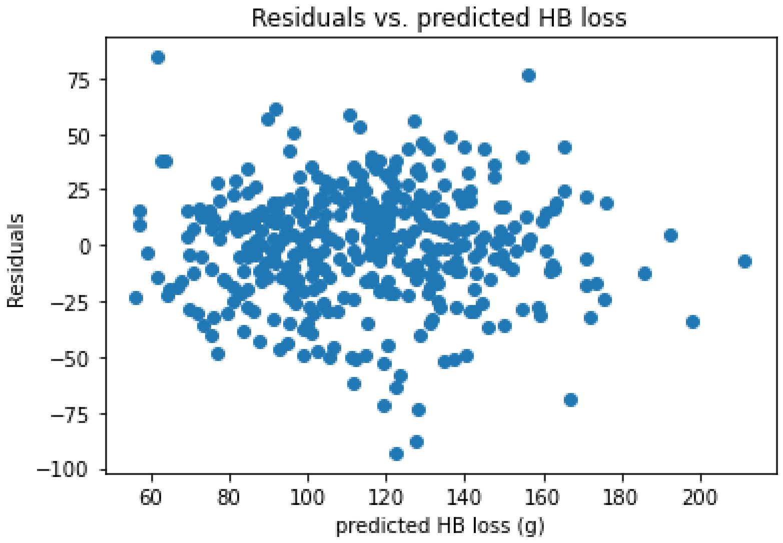

Residual vs Fitted

In [10]:
```
fig = sm.graphics.plot_partregress_grid(est)
fig.tight_layout(pad=1.0)
```

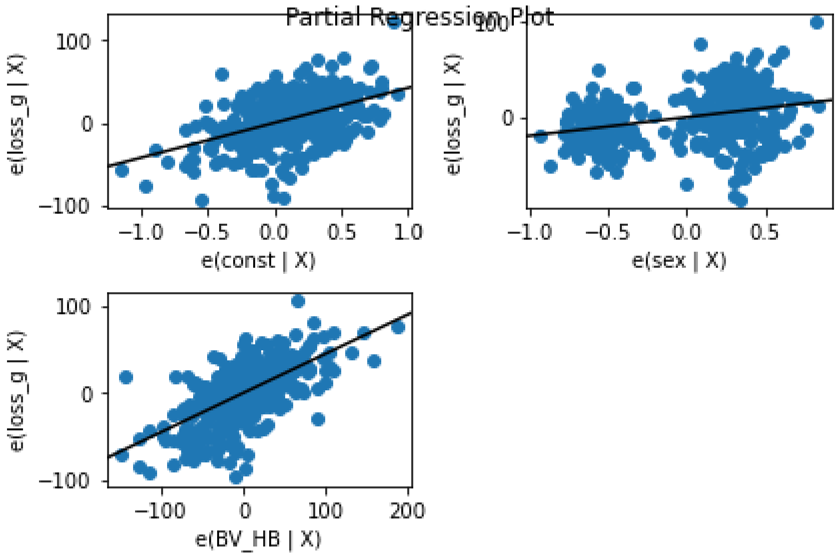

In [11]:
```
fig = sm.graphics.plot_ccpr(est, 'BV_HB')
fig.tight_layout(pad=1.0)
```

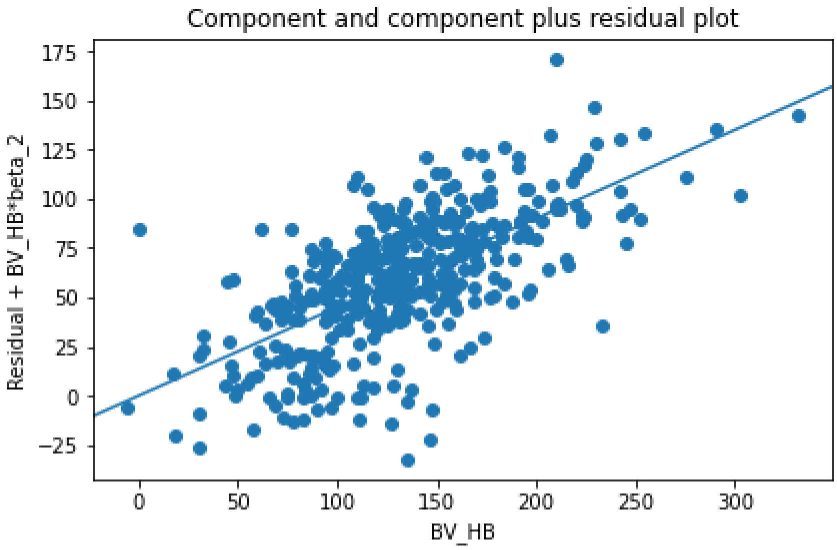

In [12]:
```
name = ['Lagrange multiplier statistic', 'p-value',
        'f-value', 'f p-value']
test = sms.het_breuschpagan(est.resid, est.model.exog)
print('Homoscedasticity: \n {}'.format(lzip(name, test)))
```

```
Homoscedasticity: 
 [('Lagrange multiplier statistic', 21.453052768590968), ('p-value', 2.1
95476441654187e-05), ('f-value', 11.246664986991272), ('f p-value', 1.77
15228558555995e-05)]
```

Q-Q-Plot

In [13]:
```
res = est.resid
fig = sm.qqplot(res, fit=True, line='45')
plt.title('QQ plot')
plt.show()
```

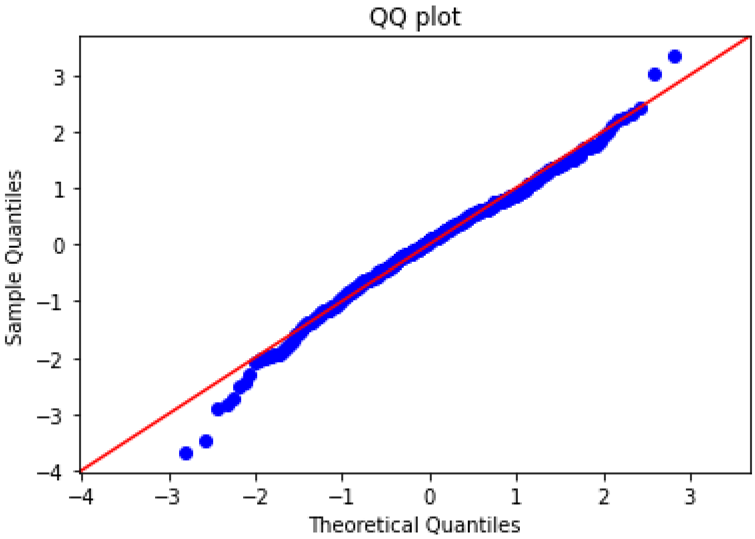

In [14]:
```
name = ['Jarque-Bera', 'Chi^2 two-tail prob.', 'Skew', 'Kurtosis']
test = sms.jarque_bera(est.resid)
print('Normal distribution of errors result: \n {}'.format(lzip(name, te
st)))
```

```
Normal distribution of errors result: 
 [('Jarque-Bera', 17.110707499444647), ('Chi^2 two-tail prob.', 0.000192
```

```
51167634807474), ('Skew', -0.3072131816742088), ('Kurtosis', 3.802505464
223569)]
```

Leverage-Resid2 Plot

In [15]:
```
fig = sm.graphics.plot_leverage_resid2(est)
fig.tight_layout(pad=1.0)
```

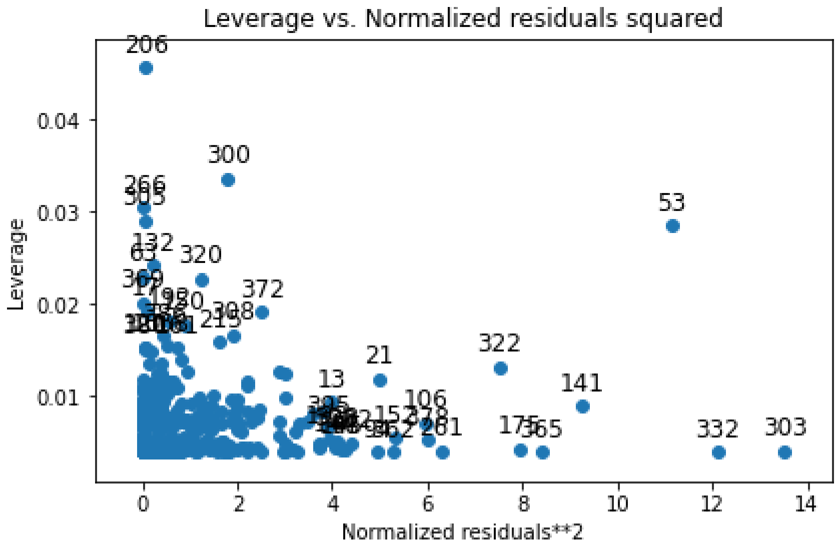

Influence plot

In [16]:
```
fig = sm.graphics.influence_plot(est)
fig.tight_layout(pad=0.0)
```

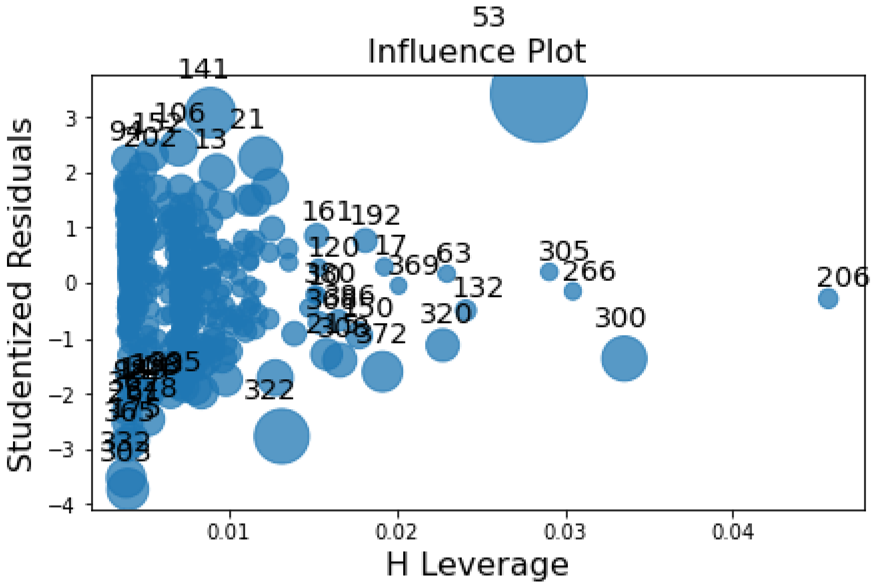

Influence statistics

In [17]:
```
influence = est.get_influence()
influence.summary_frame()
```

Out[17]:

	dfb_const	dfb_sex	dfb_BV_HB	cooks_d	standard_resid	hat_diag	dffits_internal	s

0	0.014639	0.027456	-0.018029	0.000582	0.604883	0.004747	0.041773
1	0.012960	0.052790	-0.015961	0.002463	1.356685	0.003999	0.085961
2	0.000184	0.002850	-0.000227	0.000008	0.080067	0.003869	0.004990
3	0.005529	0.011899	-0.006809	0.000112	0.272801	0.004483	0.018306
4	0.014727	-0.031177	-0.018138	0.001645	-1.089991	0.004137	-0.070250
...	...	...	...	...	...	...	...
397	-0.027722	0.020996	0.034143	0.001589	0.963122	0.005112	0.069035
398	0.051261	-0.044326	-0.063133	0.006153	-1.933129	0.004915	-0.135861
399	0.004637	-0.001013	-0.005711	0.000021	-0.088915	0.007963	-0.007966
400	0.001151	0.004234	-0.001417	0.000016	0.107629	0.004034	0.006850
401	-0.020634	0.022156	0.025412	0.001276	0.905700	0.004645	0.061871

402 rows × 9 columns

Robust regression

In [18]:
```
data_all = X_all2
data_all['blood_loss'] = y
```

In [19]:
```
est_3 = ols('blood_loss ~ sex + BV_HB', data=data_all).fit(cov_type='HC
3')
print(est_3.summary())
```

```
                            OLS Regression Results                            
==========================================================================
======
Dep. Variable:             blood_loss   R-squared:                       0
.532
Model:                            OLS   Adj. R-squared:                  0
.530
Method:                 Least Squares   F-statistic:                     2
51.8
Date:                Thu, 01 Oct 2020   Prob (F-statistic):           1.8
7e-71
Time:                        11:19:18   Log-Likelihood:                -18
69.7
No. Observations:                 402   AIC:                             3
745.
Df Residuals:                     399   BIC:                            37
57.
Df Model:                           2                                         
Covariance Type:                  HC3                                         
==========================================================================
======
                coef    std err          z      P>|z|      [0.025      0.
975]
```

```
------------------------------------------------------------------------------
----------
Intercept     42.2123      3.668     11.509      0.000      35.024      49
.401
sex           19.5549      2.720      7.189      0.000      14.223      24
.886
BV_HB          0.4505      0.030     14.911      0.000       0.391       0
.510
==============================================================================
======
Omnibus:                       13.294   Durbin-Watson:                   2
.197
Prob(Omnibus):                  0.001   Jarque-Bera (JB):               17
.111
Skew:                          -0.307   Prob(JB):                     0.00
0193
Kurtosis:                       3.803   Cond. No.
408.
==============================================================================
======

Warnings:
[1] Standard Errors are heteroscedasticity robust (HC3)
```

In [20]:

```
est_stable = rlm('blood_loss ~ sex + BV_HB', data=data_all, M=sm.robust
.norms.TukeyBiweight(3)).fit(conv='weights')
print(est_stable.summary())
```

```
                    Robust linear Model Regression Results

==============================================================================
======
Dep. Variable:             blood_loss   No. Observations:
402
Model:                            RLM   Df Residuals:
399
Method:                          IRLS   Df Model:
2
Norm:                   TukeyBiweight

Scale Est.:                       mad

Cov Type:                          H1

Date:                Thu, 01 Oct 2020

Time:                        11:19:18

No. Iterations:                    34

==============================================================================
======
                coef    std err          z      P>|z|      [0.025      0.
975]
------------------------------------------------------------------------------
----------
Intercept     43.7054      3.818     11.447      0.000      36.222      51
.189
sex           23.6331      2.979      7.934      0.000      17.795      29
.471
BV_HB          0.4367      0.029     15.067      0.000       0.380       0
```

```
.494
==============================================================================
======

If the model instance has been used for another fit with different fit p
arameters, then the fit options might not be the correct ones anymore .
```

Prediction interval

Male patients

In [48]:
```
lower = 0
upper = 350

BV_HB = np.arange(lower,upper)
const = np.array([1.0]*(upper-lower))
sex = np.array([1]*(upper-lower))

df_male_pred = pd.DataFrame({'const': const, 'sex': sex, 'BV_HB': BV_HB
})
```

In [50]:
```
ci_interval = 0.9

df_tot = X_all2
df_tot['loss'] = y
df_male = df_tot[df_tot['sex']==1]

pred_male = est_3.get_prediction(df_male_pred)
pred_male.summary_frame(alpha=1-ci_interval)
```

Out[50]:

	mean	mean_se	mean_ci_lower	mean_ci_upper	obs_ci_lower	obs_ci_upper
0	61.767283	4.975358	53.583547	69.951019	19.148601	104.385964
1	62.217798	4.947080	54.080576	70.355020	19.608024	104.827572
2	62.668313	4.918824	54.577567	70.759059	20.067390	105.269236
3	63.118828	4.890592	55.074519	71.163136	20.526700	105.710956
4	63.569343	4.862384	55.571432	71.567253	20.985954	106.152732
...	...	...	...	...	...	...
345	217.194960	6.024088	207.286216	227.103703	174.211688	260.178232
346	217.645475	6.053010	207.689159	227.601790	174.651211	260.639738
347	218.095990	6.081944	208.092082	228.099897	175.090680	261.101299
348	218.546505	6.110890	208.494984	228.598025	175.530095	261.562915
349	218.997020	6.139849	208.897866	229.096173	175.969454	262.024585

350 rows × 6 columns

In [49]:
```
ci_interval = 0.9

df_tot = X_all2
df_tot['loss'] = y
df_male = df_tot[df_tot['sex']==1]
```

```
pred_male = est_3.get_prediction(df_male_pred)
pred_result_male = pred_male.summary_frame(alpha=1-ci_interval)

plt.scatter(df_male['BV_HB'], df_male['loss'])
plt.plot(pred_result_male['mean'], color='red', label = 'prediction')
plt.plot(pred_result_male['obs_ci_lower'], color='black', label = '90%
prediction interval')
plt.plot(pred_result_male['obs_ci_upper'], color='black')
plt.title("90% prediction interval for " + r"$\it{" + 'male' + "}$" + "
patients")
plt.xlabel('Blood volume (Nadler) * HB_diff')
plt.ylabel('HB loss (g)')
plt.legend(loc="best")
```

Out[49]: <matplotlib.legend.Legend at 0x172d4f10>

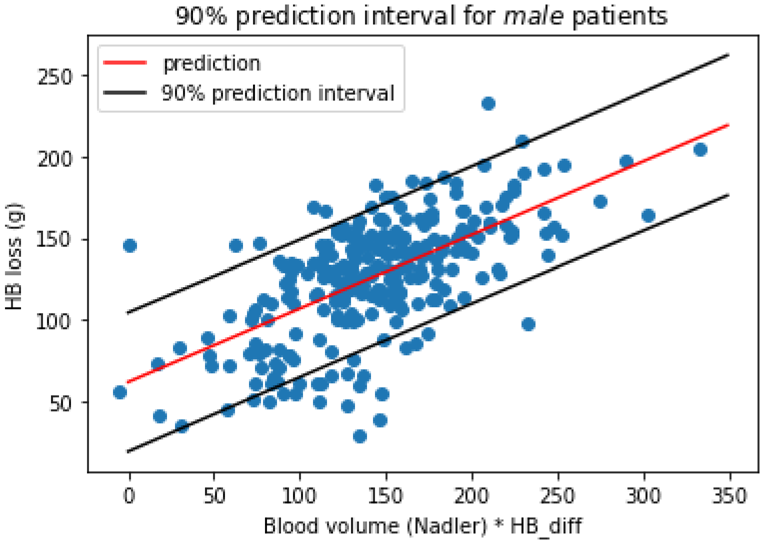

female patients

In [126]:
```
lower = 0
upper = 250

BV_HB = np.arange(lower,upper)
const = np.array([1.0]*(upper-lower))
sex = np.array([0]*(upper-lower))

df_female_pred = pd.DataFrame({'const': const, 'sex': sex, 'BV_HB': BV_
HB})
```

In [127]:
```
ci_interval = 0.9

df_tot = X_all2
df_tot['loss'] = y
df_female = df_tot[df_tot['sex']==0]

pred_female = est_3.get_prediction(df_female_pred)
pred_result_female = pred_female.summary_frame(alpha=1-ci_interval)

plt.scatter(df_female['BV_HB'], df_female['loss'])
plt.plot(pred_result_female['mean'], color='red', label = 'prediction')
plt.plot(pred_result_female['obs_ci_lower'], color='black', label = '90
% prediction interval')
```

```
plt.plot(pred_result_female['obs_ci_upper'], color='black')
plt.title("90% prediction interval for " + r"$\it{" + 'female' + "}$" +
" patients")
plt.xlabel('Blood volume (Nadler) * HB_diff')
plt.ylabel('HB loss (g)')
plt.legend(loc="best")
```

Out[127]: <matplotlib.legend.Legend at 0x18c39700>

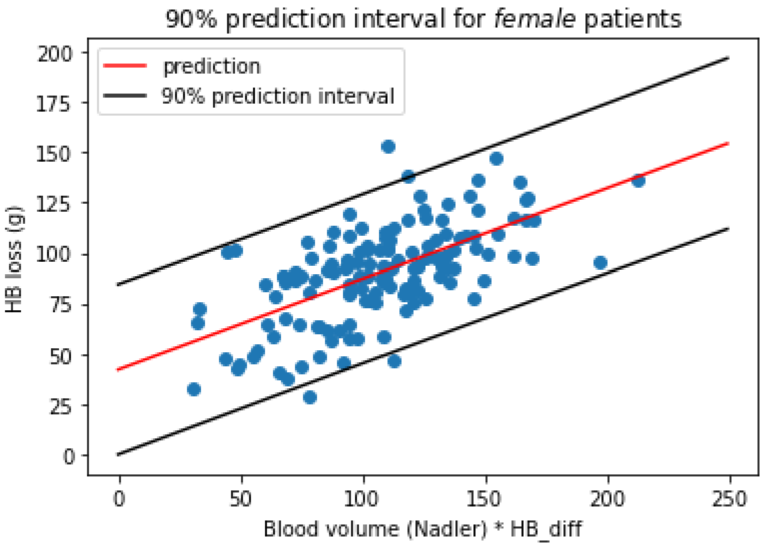

In [ ]:
